# Developing Assessments for Key Stakeholders in Pediatric Congenital Heart Disease: Qualitative Pilot Study to Inform Designing of a Medical Education Toy

**DOI:** 10.2196/63818

**Published:** 2025-01-27

**Authors:** Neda Barbazi, Ji Youn Shin, Gurumurthy Hiremath, Carlye Anne Lauff

**Affiliations:** 1 Department of Design Innovation, College of Design University of Minnesota, Twin Cities Minneapolis, MN United States; 2 Division of Cardiology, Department of Pediatrics Medical School University of Minnesota, Twin Cities Minneapolis, MN United States

**Keywords:** assessment, congenital heart disease, children health literacy, health education, caregiving, patient-centered care, design, qualitative pilot, children health, educational interventions

## Abstract

**Background:**

Congenital heart disease (CHD) is a birth defect of the heart that requires long-term care and often leads to additional health complications. Effective educational strategies are essential for improving health literacy and care outcomes. Despite affecting around 40,000 children annually in the United States, there is a gap in understanding children’s health literacy, parental educational burdens, and the efficiency of health care providers in delivering education.

**Objective:**

This qualitative pilot study aims to develop tailored assessment tools to evaluate educational needs and burdens among children with CHD, their parents, and health care providers. These assessments will inform the design of medical education toys to enhance health management and outcomes for pediatric patients with CHD and key stakeholders.

**Methods:**

Through stakeholder feedback from pediatric patients with CHD, parents, and health care providers, we developed three tailored assessments in two phases: (1) iterative development of the assessment tools and (2) pilot testing. In the first phase, we defined key concepts, conducted a literature review, and created initial drafts of the assessments. During the pilot-testing phase, 12 participants were recruited at the M Health Fairview Pediatric Specialty Clinic for Cardiology—Explorer in Minneapolis, Minnesota, United States. We gathered feedback using qualitative methods, including cognitive interviews such as think-aloud techniques, verbal probing, and observations of nonverbal cues. The data were analyzed to identify the strengths and weaknesses of each assessment item and areas for improvement.

**Results:**

The 12 participants included children with CHD (n=5), parents (n=4), and health care providers (n=3). The results showed the feasibility and effectiveness of the tailored assessments. Participants showed high levels of engagement and found the assessment items relevant to their education needs. Iterative revisions based on participant feedback improved the assessments’ clarity, relevance, and engagement for all stakeholders, including children with CHD.

**Conclusions:**

This pilot study emphasizes the importance of iterative assessment development, focusing on multistakeholder engagement. The insights gained from the development process will guide the creation of tailored assessments and inform the development of child-led educational interventions for pediatric populations with CHD.

## Introduction

### Background

Congenital heart disease (CHD) is a heart abnormality present at birth, requiring intensive medical care and often leading to life-threatening complications [1]. It affects around 40,000 newborns annually in the United States and 1% globally, posing lifelong challenges for children, families, and health care systems [1-4]. Despite significant advancements in diagnosis and treatment, CHD remains a chronic condition that demands ongoing care and specialized educational resources [5,6]. Tailored education is critical for addressing the needs of children with CHD, providing accessible educational support for parents, and equipping health care providers with efficient educational strategies. However, no tools currently exist to understand children’s health literacy, assess parental educational burdens, or measure health care providers’ efficiency in delivering education [7,8].

Children with CHD face unique barriers in understanding their condition due to developmental challenges, such as difficulty grasping abstract medical concepts or relating them to their lived experiences. These limitations can hinder their ability to adhere to treatment plans, actively participate in care, or respond effectively during emergencies and transitions to adult care [4,6,9-11]. Despite these challenges, pediatric patients are often excluded from health literacy efforts, as most resources are designed for parents or caregivers. This exclusion highlights the critical need for age-appropriate tools that empower children to engage with their care actively, ultimately improving adherence and long-term health outcomes [7,12-16].

Parents, meanwhile, bear a significant educational burden. They must interpret medical jargon, simplify it for their child, and act as intermediaries with health care providers, all while managing the emotional strain of caregiving and the cognitive load of understanding complex medical information. This burden can increase stress, reduce caregiving effectiveness, and impact family well-being. Low parental health literacy further compounds these challenges, as it is linked to medical errors and poorer health outcomes for children [17-24]. Furthermore, health care providers face the challenge of balancing clear communication with time and resource constraints, often struggling to deliver education tailored to the needs of both children and parents [11,25-27]. Addressing these gaps is essential for improving communication, care coordination, and health outcomes for children with CHD and their families [7,28].

### Study Objectives

As part of a multiphase research project, this pilot study aims to close these gaps by developing tailored assessment tools for key stakeholders in pediatric CHD education. This research comprises two phases: (1) iterative development of the assessment tools and (2) pilot testing to create, refine, and validate 3 CHD-specific assessments based on stakeholder feedback in a real-world setting. We gathered feedback using cognitive interviews and observations to assess each assessment item [29-34]. We iteratively developed face-to-face and computer-based assessments based on this feedback from all stakeholders, including children. The assessments measure changes in children’s knowledge, parental educational burdens, and health care provider efficiency before and post interventions. The results show that developing and refining these assessments are essential before introducing our medical education toy. The objectives of this study are 3-fold:

1. To assess changes in knowledge of children with CHD before and after educational interventions to empower pediatric engagement in health care innovation.
2. To measure the educational burden experienced by parents before and after interventions, focusing on their needs and challenges in navigating CHD-related educational responsibilities.
3. To assess the efficiency and effectiveness of health care providers in communicating essential information to children with CHD and their families to improve care coordination and patient outcomes.

### Prior Work

#### Assessments Developed for Pediatric Patients With CHD, Parents, and Health Care Providers

Assessments are essential for evaluating the health status and practices of pediatric patients, their parents, and health care providers across various domains [35-37]. These domains include health-related quality of life, emotional well-being, physical or psychological health, social support, and behavioral problems. For pediatric patients aged 8-18 years, assessments often involve parent proxies completing tools for younger children [38-43]. Commonly used tools in this context include the Pediatric Quality of Life Inventory (PedsQL), Pediatric Cardiac Quality of Life Inventory (PCQLI) [44-47], Child Health Questionnaire (CHQ), and Child Behavior Checklist (CBCL). However, there is a notable gap in health literacy measures for children younger than 9 years [48]. While some tools, such as Food Label Literacy for Applied Nutrition Knowledge (FLLANK) questionnaire [49], exist, none are specifically tailored to disease populations such as those with CHD.

Parental well-being and caregiving burden assessments are also critical for understanding and supporting effective caregiving. However, the specific burden related to education remains underexplored. Existing tools, such as the Parenting Stress Index/Parental Stress Scale (PSI/PSS) and the Family Impact Module (FIM) of the PedsQL, focus on parental stress, caregiving difficulty, and family functioning [50-53]. Similarly, tools such as the Caregiver Health Self-Assessment, which aims to improve the caregiver-provider dyadic relationship (commonly for older adult patients), do not address the educational needs of caregivers of patients with CHD [54-56].

For health care providers, efficiency assessments—measuring the ability to minimize wasted time and maximize outcomes—remain an ongoing challenge despite progress in evaluating physician and hospital care effectiveness [57,58]. While tools such as Key Performance Indicators (KPIs), Lean Six Sigma (LSS), and Performance Metrics are widely used to evaluate provider and hospital performance, their primary focus is on patient care outcomes (eg, patient satisfaction). These methods often overlook the educational challenges faced by health care providers themselves [59-63]. Addressing these gaps can enhance our understanding of the needs and well-being of CHD stakeholders, including children, enabling the development of more targeted and effective educational interventions. More details about these assessments are available in Multimedia Appendix 1.

#### Empowering Pediatric Engagement in Health Care Interventions

Engaging children effectively in health care interventions and assessments is challenging due to their unique developmental hurdles [64-67]. Younger children face difficulties participating because of limited attention spans and cognitive abilities, requiring reliance on parental feedback as a proxy for designing and testing interventions [68,69]. To address these challenges, studies suggest using interviews, focus groups, and activity-based methods [70-74].

Prior studies in design, human-computer interaction, and health care domains indicate that adolescents aged 13-17 years engage well in interviews and focus groups. In contrast, younger children, particularly those aged 4-12 years, benefit more from creative techniques such as activity-based methods [72-78]. Furthermore, studies highlight the importance of social factors, such as ongoing support, in alleviating children’s potential stress during research activities [79]. Empowering children through age-appropriate strategies, coupled with family facilitation, enhances their sense of ownership in health care decision-making. These approaches not only improve engagement but also strengthen the research process by addressing children’s developmental needs and promoting their active participation [79-83].

## Methods

We developed and refined 3 CHD-specific assessments to evaluate children’s knowledge, parental educational burdens, and health care provider efficiency in delivering education. This process included defining key concepts, conducting a literature review, and designing initial assessment tools. These assessments were subsequently pilot-tested with stakeholders at the M Health Fairview Pediatric Specialty Clinic for Cardiology—Explorer in Minneapolis, Minnesota, United States, to ensure validity and relevance.

### Assessment Development

#### CHD Health Literacy Children Assessment

We developed the CHD Health Literacy Children Assessment (CHD-HLCA) to evaluate CHD health literacy in children aged 4-10 years through pre- and posteducational intervention. This tool draws inspiration from the FLLANK questionnaire [84] and incorporates storytelling techniques to enhance engagement [85]. The assessment consists of 10 questions featuring simple black and white icons without color or intricate details to reduce visual distractions and avoid potential psychological and physiological influences of color. Instead, children use colored markers to answer, fostering their engagement and enthusiasm during the assessment process [86,87].

**Figure 1:**
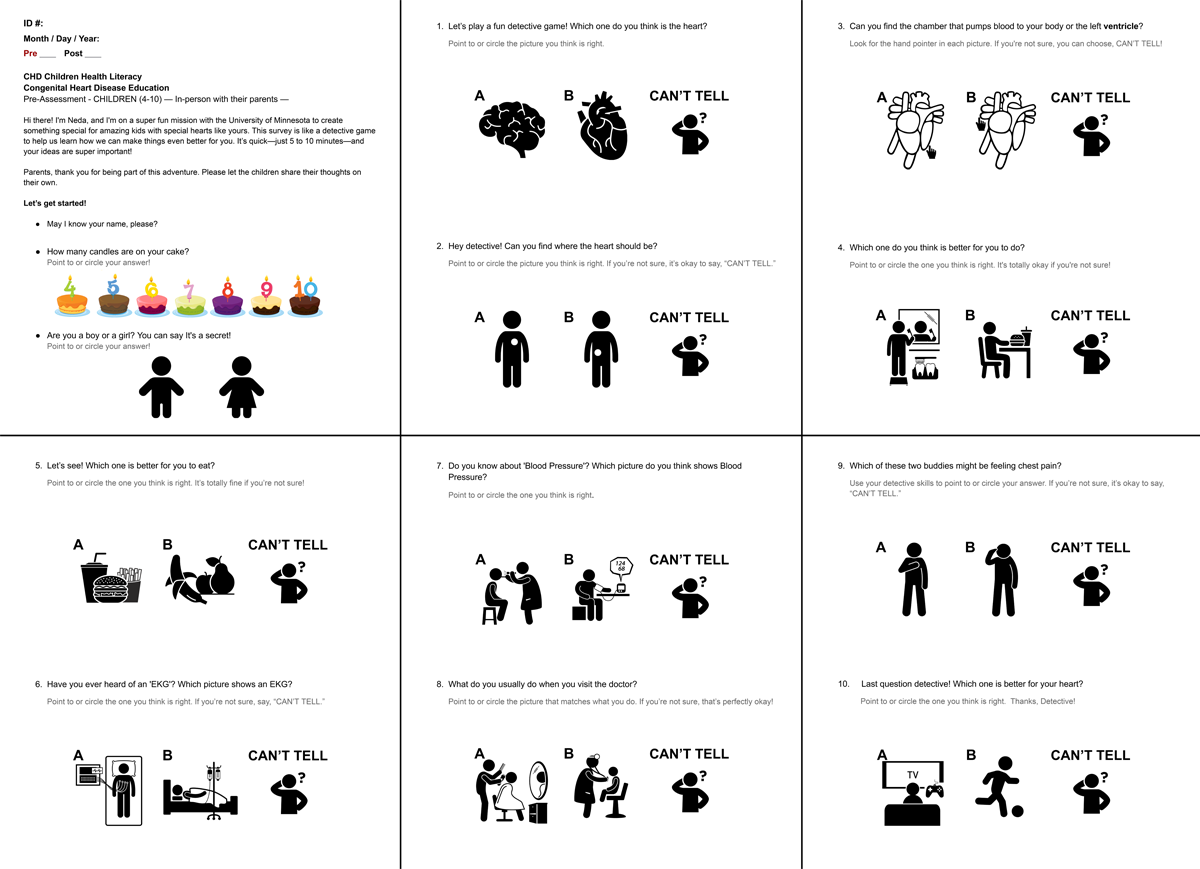
Final children’s assessment, inspired by the Food Label Literacy for Applied Nutrition Knowledge (FLLANK) questionnaire [84].

This self-report assessment is administered with the assistance of the research team or parents, particularly for younger children. The assessment covers various knowledge dimensions, including understanding CHD, preparing for doctor visits, and practicing self-care [49]. Questions offer 2 comparison options to minimize complexity, using simple icons and illustrations to reduce distractions and prevent cognitive overload. A “can’t tell” option is also included to ensure that children feel comfortable expressing uncertainty. Furthermore, a Likert scale question with Smiley Face Likerts assesses their general knowledge about their heart [88], a common method for children’s surveys. The assessment is conducted face-to-face with parental involvement, fostering a supportive environment where children can freely express themselves through drawing, crafting, or pointing to icons. It takes approximately 5-10 minutes per child and evaluates 4 key domains: Conceptual Knowledge, Comprehension, Appraisal, and Application/function specific to CHD (Figure 1 [84]).

#### CHD Parental Educational Burden Assessment

The CHD Parental Educational Burden Assessment (CHD-PEBA) is a 30-question assessment designed to evaluate parental educational responsibilities. It uses Likert scales, multiple-choice questions, matrix format, and open-ended responses to gather both quantitative and qualitative data on parental challenges. Inspired by Neuro-QoL and the Caregiver Health Self-Assessment Questionnaire [54,89,90], this self-report assessment examines parental understanding, coping mechanisms, and support needs in educating children about CHD. The assessment evaluates parents’ knowledge of CHD, their perception of their children’s understanding of the condition, and their confidence and stress levels in providing educational support. It also explores preferred information sources, information-seeking behaviors, and the time and effort parents dedicate to educating their children. Demographic data are collected to contextualize responses. Administered on the web, the survey takes approximately 5-10 minutes to complete. It serves as both a pre-and postintervention tool, measuring changes in parental educational burden over time.

#### CHD Healthcare Provider Educational Efficiency Assessment

The self-reported CHD Healthcare Provider Educational Efficiency Assessment (CHD-HEEA) health care providers’ assessment is a 20-question self-report assessment to evaluate health care providers’ educational efficiency in educating children with CHD and their families. It assesses the practices of pediatric cardiologists, fellows, nurses, and child life specialists through various question formats, including Likert scales, matrix questions, and multiple-choice and open-ended responses. Inspired by tools such as the Physician Task Checklist and Lean Six Sigma [60,63,91], the assessment examines educational practices, challenges, and strategies. Key areas of evaluation include the time and effort spent on educating children with CHD and families, perceptions of current educational methods, use of preappointment materials and self-education resources, encouragement of questions from caregivers and children, and strategies to streamline education while maintaining information accessibility. It also collects some demographic data to provide context for the responses. Administered on the web, the survey takes approximately 5-10 minutes to complete. It serves as a pre- and postassessment tool to evaluate changes in health care provider practices after educational interventions to optimize care coordination and patient education. Additional details about the 3 assessments are provided in Multimedia Appendix 2.

### Pilot Testing of Assessment Tools

During the pilot phase, we tested 3 different assessments with CHD stakeholders at the M Health Fairview Pediatric Specialty Clinic for Cardiology—Explorer.

#### Study Population

The study defined specific criteria for each stakeholder group. Children with CHD were required to be between 4 and 10 years of age, diagnosed by a health care provider, and fluent in English. Parents or guardians had to speak English and be confirmed as caregivers by a health care provider. Health care providers must be actively involved in CHD care and fluent in English.

Participants in this pilot study included children with CHD (n=5), their parents (n=4), and health care providers (n=3). Among the children were four 7-year-olds (3 males and 1 female) and one 5-year-old female (n=5). The parental and caregiver group comprised 5 mothers aged 25-44 years, including 2 White/Caucasian and 3 African American/Black participants, all with bachelor’s degrees and reporting middle to high-level incomes (n=5). However, one of the parents did not complete the assessment, resulting in a final parental group size (n=4). The health care provider group included 2 pediatric cardiology fellows and 1 physician assistant student, aged 25-44 years, with 1-5 years of experience in pediatric cardiology (n=3).

#### Study Recruitment and Informed Consent

The medical team facilitated communication with potential children with CHD and their families through purposive sampling. Parents signed parental permission forms for their children and children provided assent. We administered the children’s assessment while their parents were present but ensured that the parents did not guide their answers. After providing their own informed consent, parents received a link to complete the computer-based parental assessment. They could complete it at the clinic using the researcher’s laptop or later at home. Parents shared their experiences and provided feedback on the assessment, including its clarity and length, either during or after completion. Health care providers also participated by completing their assessments and providing feedback on their experiences. All participants received a gift card for their time. We transcribed participants’ input or feedback and anonymized results and transcripts to protect privacy.

### Data Collection

We used qualitative methods, including cognitive interviews, observational notes, and interactive techniques, to gather feedback from children, parents, and health care providers [29,30,32,34]. These methods captured participants’ impressions, behaviors, and engagement, providing rich data for iterative refinements of the assessments.

#### Logistics and Setup

To accommodate the clinic setting and time constraints, we conducted parent and child assessments simultaneously. Parents began slightly later than their children to ensure that the children were comfortable and understood the process before starting. This approach minimized parental influence on children’s responses while saving time. After completing their assessments, children engaged in painting activities, allowing parents to complete their assessments without distractions. For health care providers, assessments were scheduled flexibly, either on the web or in person, to accommodate their busy schedules.

#### Cognitive and Observational Methods

Cognitive interviews and verbal probing revealed how participants interpreted and responded to survey questions. As a qualitative method widely used in survey design, cognitive interviews helped identify ambiguities, cognitive challenges, and difficulties in understanding. Participants verbalized their thoughts concurrently (using the think-aloud technique) or retrospectively (recalling and explaining their thought processes after completing the assessment) [29-34]. Observational techniques captured nonverbal cues, such as hesitation, frustration, or excitement, along with body language and engagement levels. These observations were particularly valuable for younger children, who often struggled with abstract concepts or articulating their thoughts [76]. Combining these methods provided detailed feedback that informed refinements to our assessments.

#### Engaging Children

We used developmentally appropriate and interactive methods to engage children effectively. A researcher read questions aloud to younger children, with parents assisting if the child felt uncomfortable interacting with the research team. To make the activity engaging, we incorporated storytelling and asked children to role-play as detectives solving questions. Prompts such as “Hey, detective! Let’s figure out which, what, or where!” encouraged participation. Children used markers and craft materials, such as multicolor pom-poms, to point to their answers, making the process interactive and enjoyable. For older children who could read independently, we encouraged self-guided responses and asked clarifying questions to explore their thought processes. The think-aloud technique and verbal probing provided deeper insights into their reasoning [70-74]. To ensure that children understood the questions rather than guessing, we rephrased questions after they provided answers. For example, if a child pointed to an icon for chest pain, we asked, “Do you know where your chest is?” and used physical prompts to confirm their understanding. This approach validated their answers and often prompted children to share personal experiences, enriching the feedback [66,67,76,83]. 

#### Refining the Assessments

We iteratively refined the assessments, tailoring questions to each stakeholder group by focusing on their thoughts, understanding, relevance, completeness, survey length, and overall experience.

For the children’s assessment, we prioritized meeting the developmental needs of children aged 4-10 years based on criteria provided by health care providers. These criteria outlined essential knowledge for this age group while addressing the additional challenges faced by younger or newly diagnosed children. Testing revealed that younger children often struggled with multistep questions, so we simplified these into single, clear actions. Language and visuals were adjusted for clarity while retaining enough detail to engage older children. Older children provided feedback on question relevance and reflected on how they might have responded when younger, offering insights that shaped refinements.

Each testing round informed adjustments to ensure that the questions were clear, precise, and engaging for all stakeholders in CHD pediatric care. These assessments now support pre- and postassessment stages to validate interventions, including our ongoing study of medical educational toys for CHD pediatric populations. Table 1 outlines the development and testing process, with sample prompts provided in Multimedia Appendix 3.

**Table 1:** Iterative development and pilot testing of congenital heart disease (CHD) assessments.

Step	Description	Stakeholders involved	Outcomes
1. Define key concepts	Define health literacy, parental educational burden, and health care provider efficiency	Research team, pediatric cardiologist	Established key concepts for the assessment framework
2. Conduct literature review	Review existing best practices and assessments	Research team	Insights for developing new assessments
3. Develop initial assessments	Draft assessments for children, parents, and health care providers	Research team, pediatric cardiologist	Initial assessments ready for review
4. Pilot testing—first iteration	Test initial assessments with children with CHD, parents, and health care providers	Children with CHD, parents, and health care providers	Feedback on relevance, clarity, and appropriateness
5. Analyze feedback	Analyze feedback from pilot testing	Research team, pediatric cardiologist	Identify strengths, weaknesses, and improvements
6. Refine assessments	Modify assessments based on feedback	Research team	Improved assessments
7. Pilot testing—further iterations	Conduct additional testing and refinement	Children with CHD, parents, and health care providers	Continuous improvement and validation
8. Final analysis	Analyze all data and feedback to finalize assessments	Research team, pediatric cardiologist	Validated assessments for implementation

### Data Analysis

Our research team held weekly meetings to analyze assessment data and stakeholder feedback. Using qualitative methods, including thematic analysis, we identified common themes from feedback and observational data [92,93]. The first author coded the data to highlight relevant themes, focusing on the assessment’s reliability, engagement, and responsiveness to meet all stakeholders’ needs. During each round of pilot testing, we prioritized feedback using four criteria: (1) its potential impact on the assessment’s effectiveness, (2) alignment with intended outcomes, (3) feasibility of incorporating changes, and (4) stakeholder preferences. Feedback was categorized by feasibility, clarity, relevance to educational needs, and participant engagement, then ranked by the frequency and significance of reported issues. We implemented revisions iteratively, prioritizing aspects that most improved comprehension and ease of response. This systematic process enhanced the assessments’ accuracy and relevance across stakeholder groups. During weekly meetings, the team reviewed how each piece of feedback aligned with the established criteria and worked collaboratively to resolve discrepancies. This iterative process led to the development of a set of codes such as “strengths,” “weaknesses,” and “areas of improvement.” These ongoing discussions and thematic analyses refined the assessments and informed adjustments for subsequent pilot tests [94].

### Ethical Considerations

The study received ethical approval from the University of Minnesota institutional review board (STUDY00020670). The medical team facilitated communication with potential participants—children with CHD and their families—through purposive sampling. Informed consent was obtained from all participants: parents also signed parental permission forms for their children, and children provided assent. Participants were informed about the study’s purpose, procedures, and their right to withdraw at any time. Data were anonymized and deidentified during transcription and analysis, with all personal information securely stored in adherence to institutional guidelines. Participants received a gift card as compensation for their time, ensuring fairness and transparency.

## Results

We identified consistent themes across three assessments: (1) assessment engagement, (2) relevance and structure of assessment, and (3) opportunities of assessments. These themes guided iterative revisions before each new pilot test. In this section, we summarize the findings from (1) children’s assessments, (2) parents’ assessments, and (3) health care providers’ assessments. Some feedback from key stakeholders on 3 survey experiences can be found in Multimedia Appendix 2.

### Assessments Engagement

Both children (C) and parents (P) actively engaged in the children’s assessment process. Parents were surprised by their children’s interest and enjoyment, with one parent expressing, “I wasn’t sure she’d even talk. Wow!” It appeared that the interactive elements, such as choosing pens or markers and storytelling, not only boosted curiosity and made them concentrate on doing the assessment but also eased the anxiety and fear of children. For instance, C3 quickly transitioned from nervousness about another medical procedure to excitement upon seeing colorful markers, exclaiming, “Yay, I can paint here in the doctor’s office!” Initially, she hid under the bed due to fear when we entered the examination room. However, she relaxed and became comfortable after seeing the markers and being invited to answer the questions using different colors. Even children like C2, initially focused on their iPad, became engaged, asking, “Can I use all colors? I can answer anything like this!”

**Figure 2:**
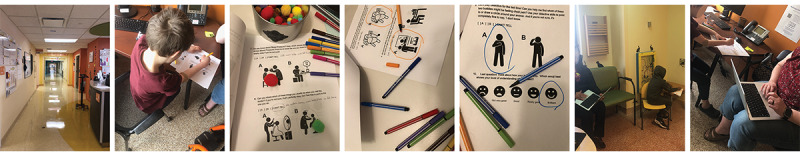
Children’s interactions with children’s assessments and parental responses to their assessments at the Explorer clinic.

Parents appreciated the playful and instructive design of the assessments, with one commenting, “It’s like teaching by itself through playtime. She’s telling me all you've asked her.” After finishing their assessments, we provided paper to children like C4, whose mother was also being assessed. This engagement prompted them to draw, becoming so engrossed to continue that they requested us to stay there even after the doctor’s arrival (Figure 2). Initially, parental influence impacted C1’s responses, but gentle interventions and prioritizing children’s uninfluenced responses over accuracy fostered independent responses in other pilot tests. Through face-to-face interaction using cognitive interviews akin to semistructured interviews [95,96], we collected less biased data from children rather than their parents, maintained attention, and reduced parental influence.

Similarly, in the clinic, parents provided feedback after their children’s assessments through a think-aloud and verbal probing method [29,32] while answering the computer-based parents’ assessment using Typeform (Typeform SL). During the assessment, P4 noted, “You know what to ask.” P3 mentioned that they would change only some of the wording. However, despite this feedback, P3 described the survey as “clear, easy, feeling good; finally, somebody asks!” This feedback highlighted the ease of use, clear directions, and meeting the participants’ needs, facilitating effective data collection. The health care providers’ assessment also received positive feedback for its engaging interface, with H1 noting, “Much better than usual surveys,” and H3 stating, “...really interactive with photos and video, and buttons. It’s easy....” Such feedback underscores the effectiveness of engaging participants and facilitating data collection.

### Relevance and Structure of Assessment

The effectiveness of engaging all stakeholders through assessments depends on their relevance and well-structured design. Maintaining high relevance across all assessments, we noticed an interesting trend involving the Smiley Face Likert question during the children’s assessment. Despite our efforts to adjust the question wording, children consistently chose the happiest face in the initial version of the assessment. They emphasized their positive feelings associated with smiley faces by saying, “I chose it because I like it!” We expected this issue but still tested the smiley face Likert question due to its common use in children’s surveys. To improve engagement and reliability, we found that comparison questions, structured like a right or wrong game format, were more effective. Children showed high concentration levels and often asked, “Is this right?” They felt like participating in a game, increasing their involvement in the assessment process. Incorporating feedback from our medical team and parents highlighting the assessment’s educational value, we replaced the Likert question with a prompt offering a choice between physical activity and screen time (Figure 1 [84]), addressing another relevant habit for children with CHD. We also adjusted the question order to observe response variations, albeit with limited reliability due to its implementation with only participants C2 and C3 immediately after the initial version.

**Figure 3:**
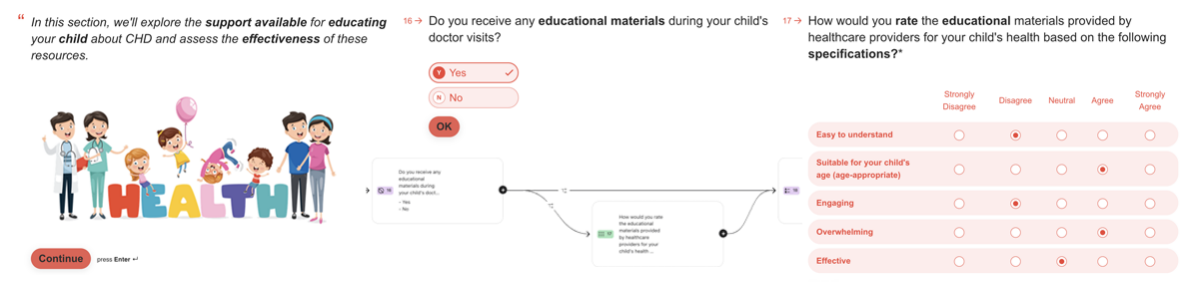
Congenital Heart Disease Parental Educational Burden Assessment (CHD-PEBA) logic model question.

P2, P3, P4, and P5 actively engaged with the questions in the parents’ assessment, finding them clear, easy, and relevant. However, P2 noted assumptions in specific questions regarding the prior receipt of educational material during doctor visits. They proposed a preliminary question to confirm whether educational material was used before assessing its appropriateness (Figure 3). They asked, “How do you know if we got any educational material before asking how good it was? It’d make more sense to check if we received any and then ask about it.” Interestingly, when explicitly asked about this question, P3, a follow-up patient, did not express the same concern. Another participant suggested improving the context of the question about comfort levels during clinic visits (follow-ups or surgeries), which we addressed in the later version. We condensed the questionnaire to 25 items in the latest parent assessments by adding a matrix question.

**Figure 4:**
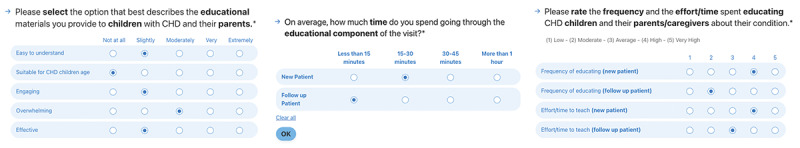
Congenital Heart Disease Healthcare Provider Educational Efficiency Assessment (CHD-HEEA) matrix questions.

In response to initial feedback on the health care providers’ assessment, H1 proposed categorizing questions differently for new and follow-up patients. All participants provided positive feedback regarding the matrix questions (Figure 4). H2 preferred to keep the existing matrix questions but suggested reordering them for better clarity and efficiency. They emphasized that combining related aspects into single questions helps respondents compare options and find the correct answer more easily: “Having one question for each of these aspects would make the questionnaire longer and harder to compare when answering; it just seems more practical this way.” Furthermore, H2 recommended adding a specific demographic—vulnerable parents—to measure parental CHD information instead of using a general category. These suggestions were integrated into version 03, reducing the length to 15 items.

### Opportunities of Assessments

We used patients’ wait time between their initial screenings and the meeting with their physician to complete the assessments. This integration not only streamlined care coordination and reduced wait times but also engaged families and alleviated worries, as noted by P4, “It keeps us good busy!” This suggests that our design intervention (an educational toy) can be implemented during patients’ wait time as well. The assessments also doubled as educational tools for children, engaging them in learning about their condition and easing anxiety. Parents found solace in sharing burdens, while health care providers gained insights into their practices and sought potential solutions. These assessments exceeded their initial purposes, offering education, support, and reflection opportunities for child-led approaches in pediatric care.

## Discussion

### Principal Results

During face-to-face interviews, we identified areas for refinement in assessment items, such as clarifying the context of health care experiences for children’s comfort-level rating or specifying the type of visit for health care providers. Observational notes highlighted instances where assumptions in specific questions, such as the prior receipt of educational material during doctor visits, caused confusion among interviewers. To alleviate this issue, we proposed preliminary questions. Despite these challenges, participants generally interpreted the majority of survey items as intended, with occasional adjustments to wording choices for better comprehension. Furthermore, we changed the order of questions based on feedback and observations to improve the structure of the assessment.

The pilot testing evaluated the feasibility and effectiveness of the tailored assessments. Iterative revisions further improved their clarity and appropriateness. We ensured the validity of the assessments through a collaborative and iterative approach, incorporating perspectives from all stakeholders. As a result, the assessments offer a comprehensive understanding of educational needs and burdens, providing valuable insights to guide the development of targeted educational interventions.

### Comparison With Prior Work

The tailored assessments developed in this pilot study address a gap in the existing literature by focusing on the educational needs of pediatric patients with CHD, their parents, and health care providers [7,8,27]. While existing assessment tools mainly measure health-related quality of life and emotional well-being, they offer limited insight into health literacy and the unique educational challenges associated with CHD [38-56]. Moreover, these assessments are primarily developed quantitatively and lack qualitative insights into educational needs and burdens. We used qualitative methods to explore how respondents interpret, understand, and respond to specific survey items. This approach offered a more comprehensive understanding of the questions that assessments should address [29-32,93-97].

Through collaboration with stakeholders, including children, we developed and refined assessments to evaluate children’s knowledge, parental educational burdens, and health care provider efficiency in pediatric CHD care. Including children in the development process was crucial due to the lack of tailored educational materials for children with CHD and the complexities involved in assessing this population [64-67,98]. While parents were present during the children’s assessments to provide comfort, their involvement was carefully managed to ensure that it did not interfere with the children’s meaningful participation. This approach contrasts with prior methods, where parents often act as proxies, potentially biasing the results [68,69]. Feedback from all stakeholders was collected post assessment to gain insights into their experiences and further improve the assessments.

We conducted pilot tests for two reasons: (1) to refine the assessments before involving more stakeholders, specifically children, and (2) to ensure the assessments were well adapted to meet all stakeholders’ needs before the actual study [99,100]. This pilot study revealed critical gaps in existing tools and advanced the methodology for developing educational assessments. Emphasizing collaborative, iterative, and direct engagement with children, parents, and health care providers ultimately leads to the design of tailored assessments. These assessments are crucial for informing the development of effective, child-led educational interventions for pediatric populations with CHD, demonstrating feasibility before broader implementation.

The assessments developed in this study have potential applications in routine clinical practice, offering health care providers a structured tool to assess and enhance CHD-related health literacy among pediatric patients and their families. By integrating these assessments into preappointment resources or waiting room activities, health care providers can identify educational gaps early and address them proactively. Furthermore, the assessments could complement existing educational interventions, providing feedback on the effectiveness of child- and family-centered resources to improve health literacy. This integration would not only inform the design of more effective child- and family-centered resources but also support continuous improvement of educational interventions. This approach aligns with broader goals in pediatric health care to support lifelong health management through early literacy interventions.

### Limitations

Although the pilot testing provided valuable insights, several limitations are acknowledged. First, despite efforts to minimize parental influence, their presence may have still impacted some children’s responses. Future research should explore strategies to reduce this influence further and ensure more independent responses from children. Second, the sample distribution was not spread evenly across age groups, with only 1 child aged 5 years and 4 children aged 7 years. This uneven distribution may affect the representativeness of the findings. Future studies should aim for a more balanced age distribution. Third, conducting assessments for both children and parents at the clinic during their visit might have influenced their responses, as they were exposed to medical procedures. Furthermore, the researcher’s presence during the assessment of parents and children could have influenced their feedback. Finally, while general accessibility was considered, specific adaptations for educational disabilities were not within the scope of this pilot study.

### Conclusions

This pilot study aims to improve educational interventions and care coordination through tailored assessments designed by stakeholder feedback, including affected children. The findings emphasize the importance of interactive and qualitative methods that foster multistakeholder engagement and ensure question relevance. These assessments can potentially enhance child-led interventions and improve outcomes for patients with CHD from childhood through to adult care. However, the iterative development process highlighted several challenges, such as managing parental presence to encourage independent responses from children and the logistical complexities of recruiting diverse participants within clinical settings. It is also important to ensure that assessments for younger children are read aloud without influencing their responses. Integrating these assessments into real-world clinical workflows requires adaptability to avoid disrupting patient care. Addressing these practical challenges will be essential to scaling and sustaining the use of these assessments in diverse health care contexts.

## Appendix

Multimedia Appendix 1 Existing assessment tools.

Multimedia Appendix 2 Three congenital heart disease–specific assessments.

Multimedia Appendix 3 Sample prompts and feedback from data collection.

## Abbreviations

CBCL: Child Behavior Checklist
CHD: congenital heart disease
CHD-HEEA: Congenital Heart Disease Healthcare Provider Educational Efficiency Assessment
CHD-HLCA: Congenital Heart Disease Health Literacy Children Assessment
CHD-PEBA: Congenital Heart Disease Parental Educational Burden Assessment
CHQ: Child Health Questionnaire
FIM: Family Impact Module
FLLANK: Food Label Literacy for Applied Nutrition Knowledge
KPI: Key Performance Indicator
LSS: Lean Six Sigma
PCQLI: Pediatric Cardiac Quality of Life Inventory
PedsQL: Pediatric Quality of Life Inventory
PSI/PSS: Parenting Stress Index/Parental Stress Scale
