# Supplementary material for: Developing Assessments for Key Stakeholders in Pediatric Congenital Heart Disease: Qualitative Pilot Study to Inform Designing of a Medical Education Toy
Source: JMIR Form Res. 2025 Jan 27;9:e63818. doi: 10.2196/63818 (PMC11811657; doi:10.2196/63818)
Supplement: Multimedia Appendix 1 [file formative_v9i1e63818_app1.docx]

## Multimedia Appendix 1

Existing assessment tools for pediatric children.

| **Assessment Tool** | **Name** | **Purpose** | **Target Population** | **Dimensions** |
| --- | --- | --- | --- | --- |
| **General Health-Related Quality of Life (HRQoL) Tools** | Pediatric Quality of Life Inventory (PedsQL) | Assess overall well-being and HRQoL in healthy children and adolescents and those with acute and chronic conditions | Children and adolescents (ages 8-12)  (self-assessment)  (proxy assessment) | Overall well-being |
|  | Child Health Questionnaire (CHQ) | Evaluate HRQoL | Children and adolescents  (aged 5-18) | HRQoL |
|  | KID-SCREEN questionnaire | Provide comprehensive assessments in both healthy and ill children and adolescents | Children and adolescents (aged 8-18)  (self-assessment)  (proxy assessment) | Physical well-being, psychological well-being, parent relations and autonomy, social support and peers, school environment |
| **Behavioral and Emotional Assessment Tools** | Child Behavior Checklist (CBCL) | Assess behavioral and emotional aspects | Children and adolescents  (aged 1.5–5)  (aged 6–18)  (self-assessment)  (proxy assessment) | Behavioral and emotional aspects |
|  | Pediatric Evaluation of Disability Inventory (PEDI) | Clinical assessment that samples key functional capabilities and performance in children | Children  (aged 6 months to 7½)  (proxy assessment) | Functional status |
|  | Children's Coping Strategies Checklist (CCSC) | Assess coping strategies | Children and adolescents (aged 8-18) | Coping strategies |
|  | Strengths and Difficulties Questionnaire (SDQ) | Evaluate behavioral strengths and difficulties | Children and adolescents  (aged 2-17)  (self-assessment)  (proxy assessment) | Behavioral strengths and difficulties |
|  | Self-Perception Profile for Children (SPPC) | Assess self-perception | Children | Self-worth, competencies, emotional states, behavioral problems |
|  | Self-Perception Profile for Adolescents (SPPA) | Assess self-perception | Adolescents | Self-worth, competencies, emotional states, behavioral problems |
| **Disease-Specific HRQoL Tools for CHD Children** | Pediatric Cardiac Quality of Life Inventory (PCQLI) | Measure disease-specific HRQoL | Children (8–12), Adolescents (13–18) | Total HRQoL, disease impact subscale, psychosocial impact subscale |
| **Health Literacy Assessment Tools for CHD Children** | European Health Literacy Questionnaire for fourth graders (HLS-Child-Q15) | Assess health literacy | Children (aged 0-9)  Adolescents (10–17) | Health literacy |
|  | An Instrument for Measuring Health Literacy in Children (QUIGK-K) | Assess health literacy | Children (aged 0-9)  Adolescents (10–17) | Health literacy |
|  | Food Label Literacy for Applied Nutrition Knowledge Questionnaire (FLLANK) | Assess food label literacy | Children (aged 0-9) | Food label literacy |
