# Supplementary material for: Developing Assessments for Key Stakeholders in Pediatric Congenital Heart Disease: Qualitative Pilot Study to Inform Designing of a Medical Education Toy
Source: JMIR Form Res. 2025 Jan 27;9:e63818. doi: 10.2196/63818 (PMC11811657; doi:10.2196/63818)
Supplement: Multimedia Appendix 2 [file formative_v9i1e63818_app2.docx]

## Multimedia Appendix 2

Developed assessment tools for CHD children, parents, and healthcare providers.

| **Assessment Tool** | **Name** | **Purpose** | **Target Population** | **Dimensions** | **Time and Mode** |
| --- | --- | --- | --- | --- | --- |
| **Health Literacy Assessment Tools for CHD Children** | Congenital Heart Disease Health Literacy Children Assessment (CHD-HLCA) | Evaluate CHD health literacy in young children | Children (ages 4-10)  (self-assessment) | Understanding the condition, doctor visit procedures, self-care practices (CHD health literacy | 5 to 10 minutes  Paper and pencil, Face-to-face |
| **Educational Burden Assessment Tools for CHD Parents/Caregivers** | Congenital Heart Disease Parental Educational Burden Assessment (CHD-PEBA) | Evaluate CHD parental educational responsibilities and support needs | Parents/caregivers of children with CHD  (children aged 4–10)  (self-assessment) | Understanding needs, preferred sources of information, information-seeking behaviors, time/effort spent educating children | 5 to 10 minutes  Computer-based |
| **Healthcare Provider Educational Efficiency Assessment for CHD** | Congenital Heart Disease Healthcare Provider Educational Efficiency Assessment (CHD-HEEA) | Assess healthcare providers' CHD educational efficiency and effectiveness | CHD Healthcare Providers  (children aged 4–10)  (self-assessment) | Educational practices, challenges, strategies, time/effort spent educating patients, perceptions of current methods | 5 to 10 minutes  Computer-based |

#### Congenital Heart Disease Health Literacy Children Assessment (CHD-HLCA)

**ID #:**

**Month / Day / Year:**

**Pre** _____ **Post** _____

**CHD Children Health Literacy**

**Congenital Heart Disease Education**

Pre-Assessment - CHILDREN (4-10) — In-person with their parents —

Hi there! I'm Neda, and I'm on a super fun mission with the University of Minnesota to create something special for amazing kids with special hearts like yours. This survey is like a detective game to help us learn how we can make things even better for you. It’s quick—just 5 to 10 minutes—and your ideas are super important!

Parents, thank you for being part of this adventure. Please let the children share their thoughts on their own.

**Let’s get started!**

- May I know your name, please?
- How many candles are on your cake?

Point to or circle your answer!


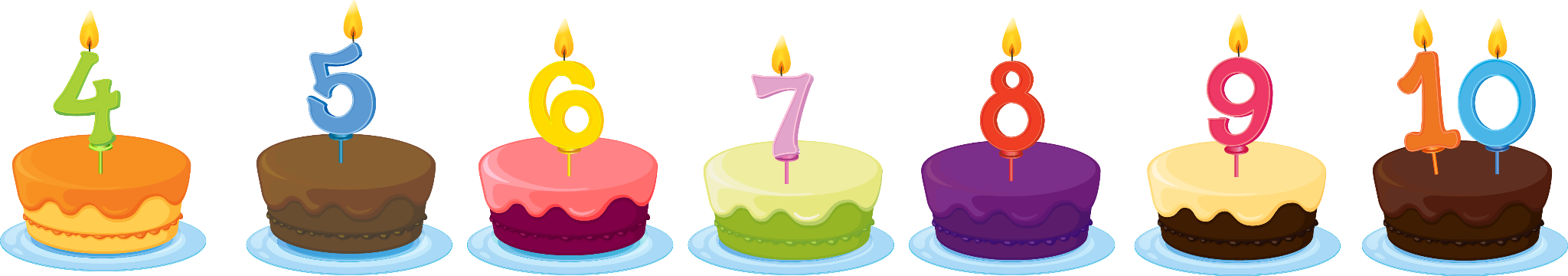


- Are you a boy or a girl? You can say It's a secret!

Point to or circle your answer!

**
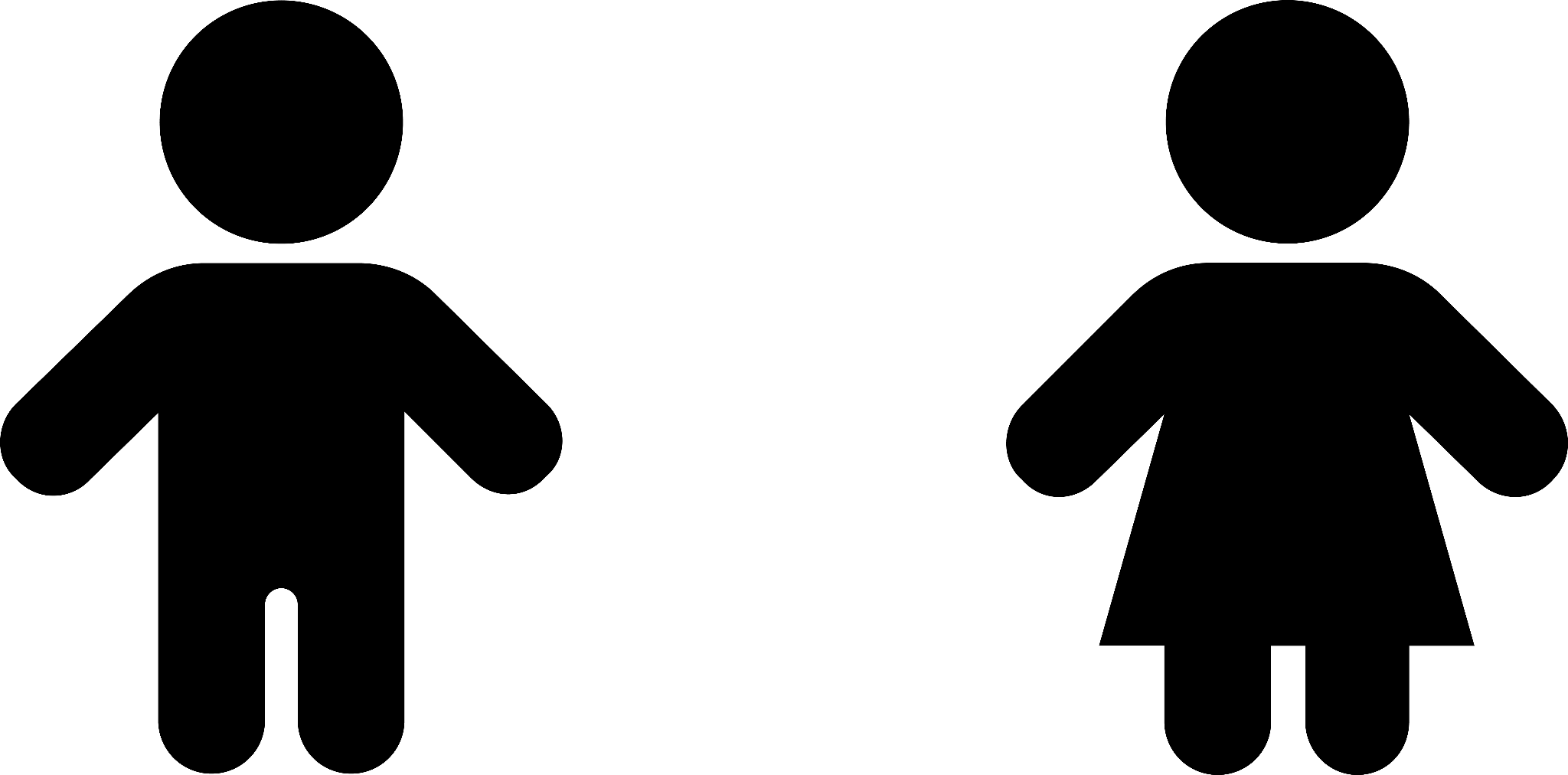
**

1. Let’s play a fun detective game! Which one do you think is the heart?

Point to or circle the picture you think is right.


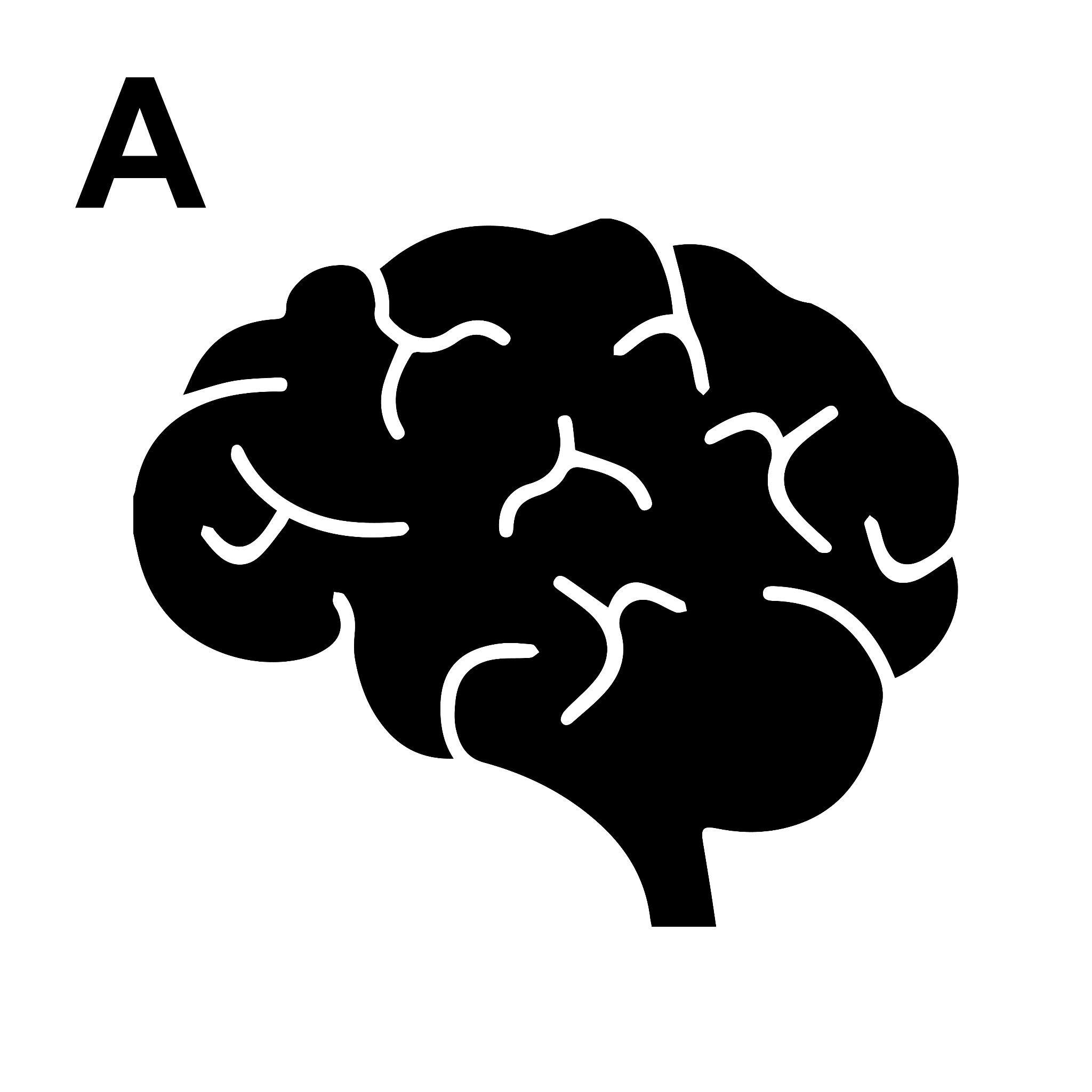

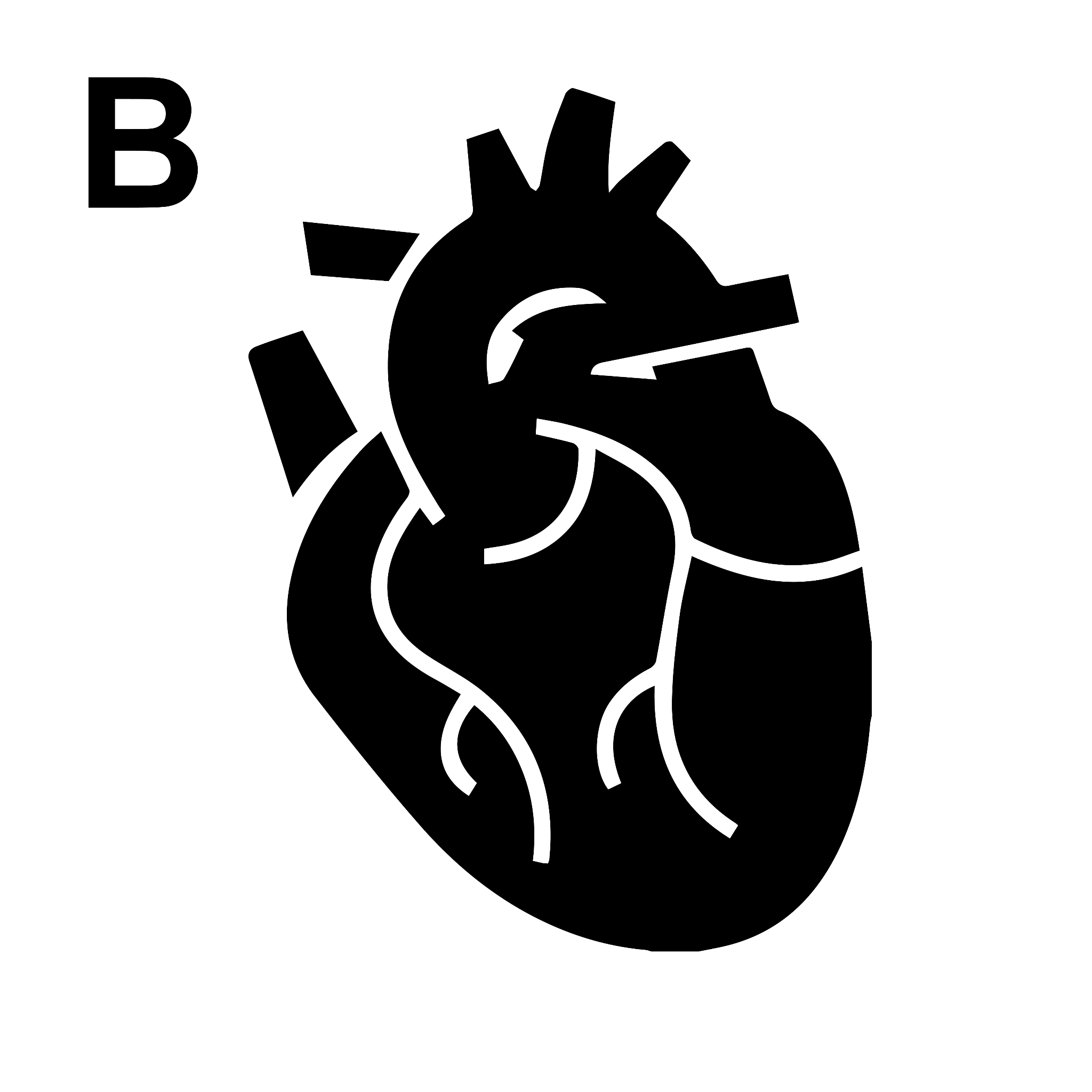
**
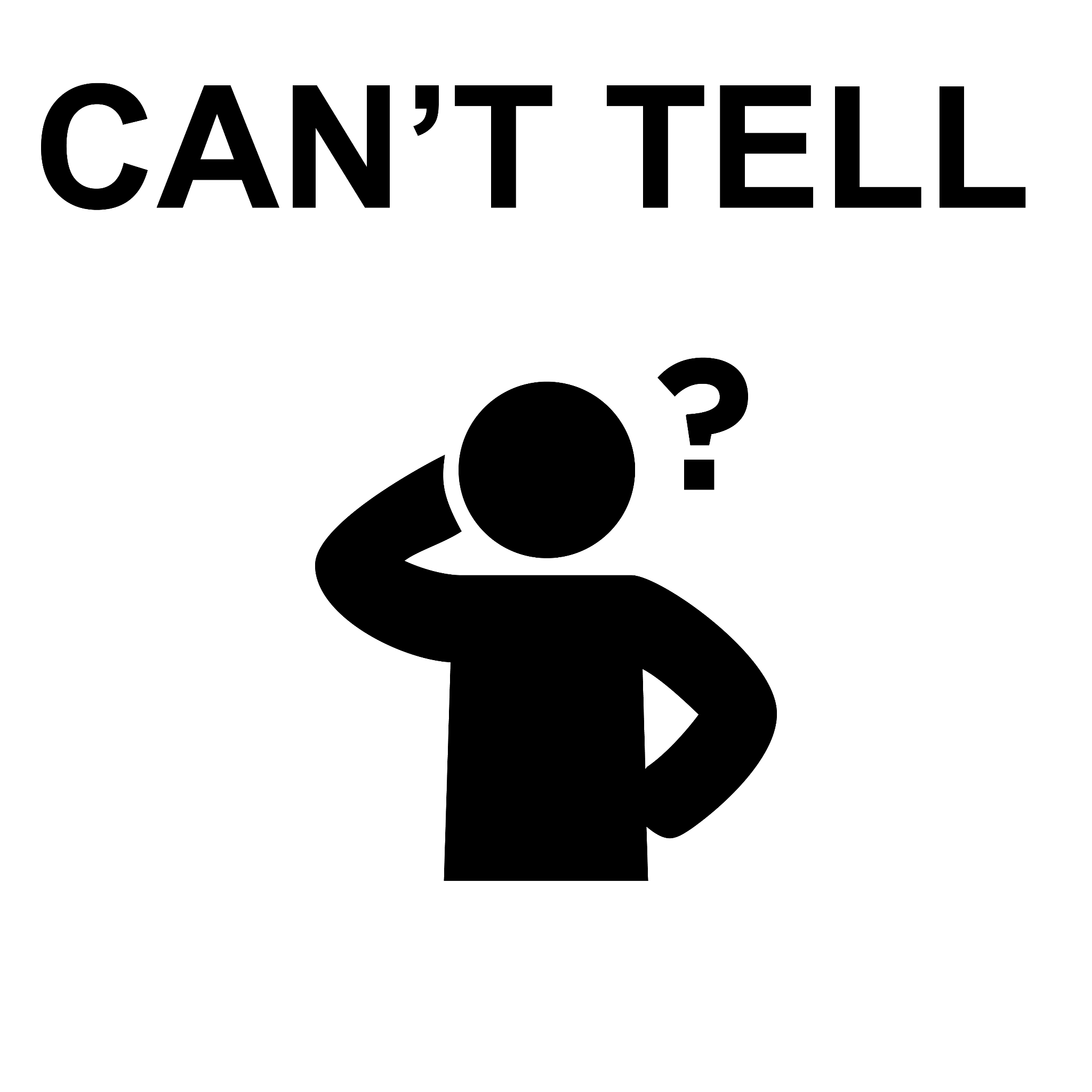
**

1. Hey detective! Can you find where the heart should be?

Point to or circle the picture you think is right. If you’re not sure, it’s okay to say, “CAN’T TELL.”


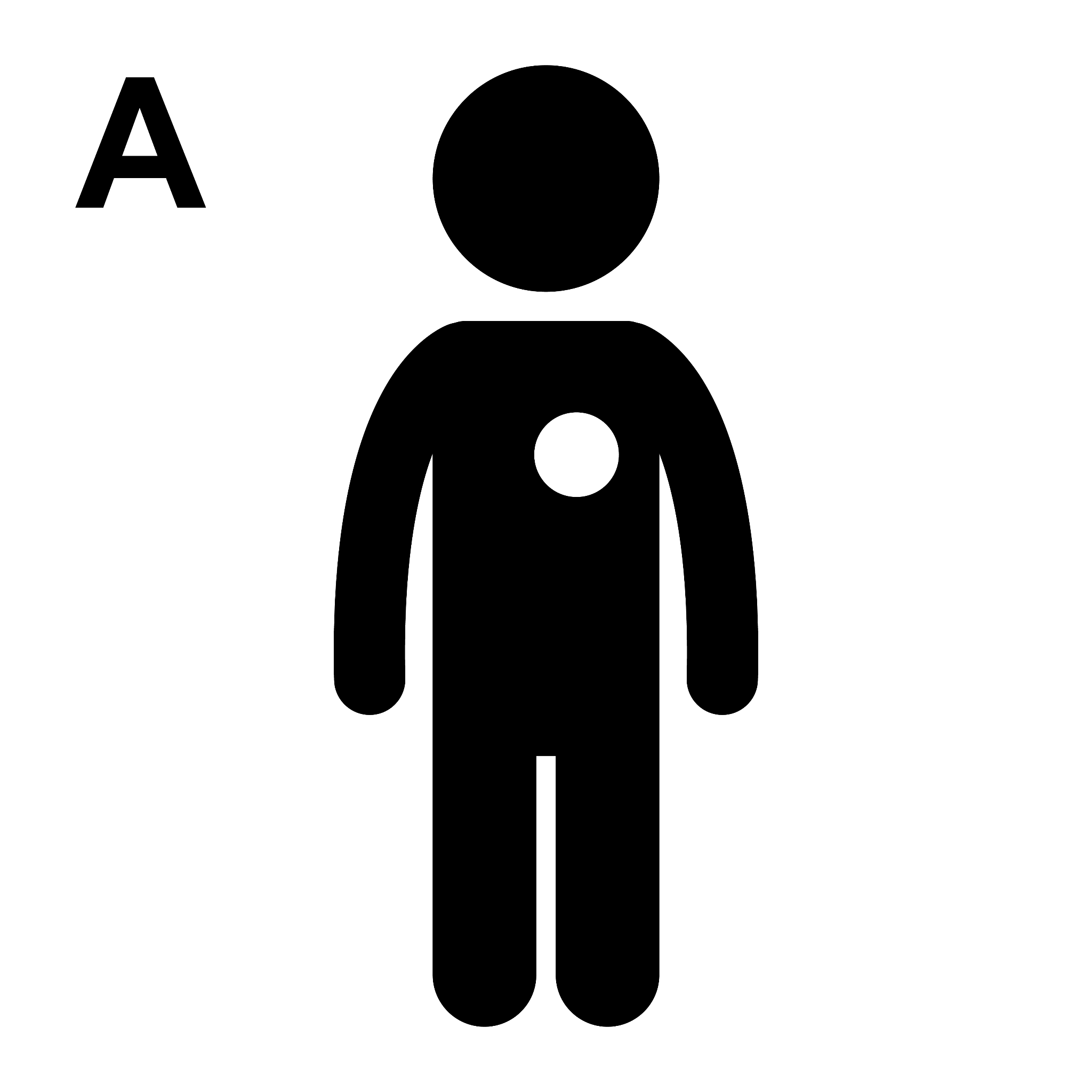

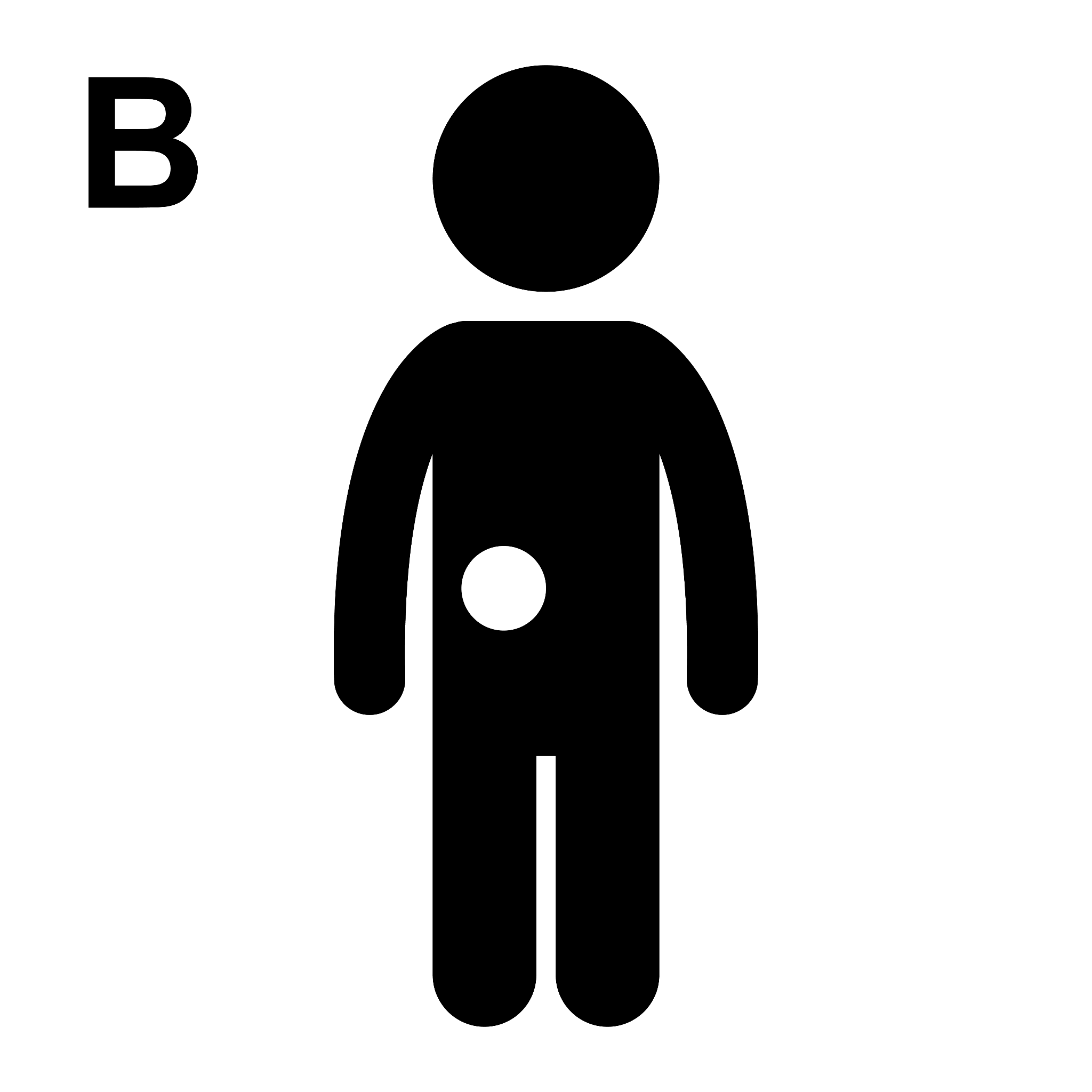
**
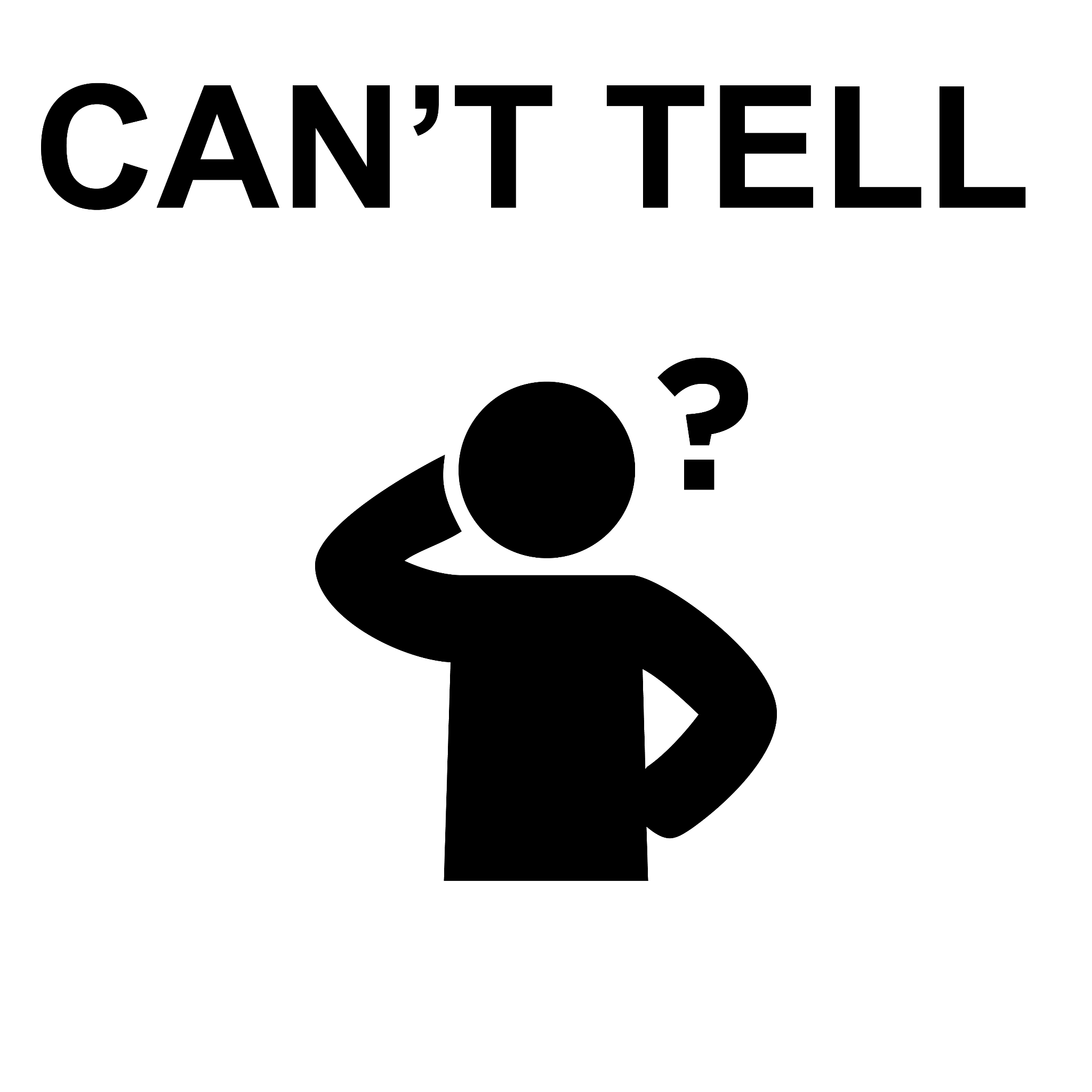
**

1. Can you find the chamber that pumps blood to your body or the left **ventricle**?

Look for the hand pointer in each picture. If you're not sure, you can choose, CAN’T TELL!


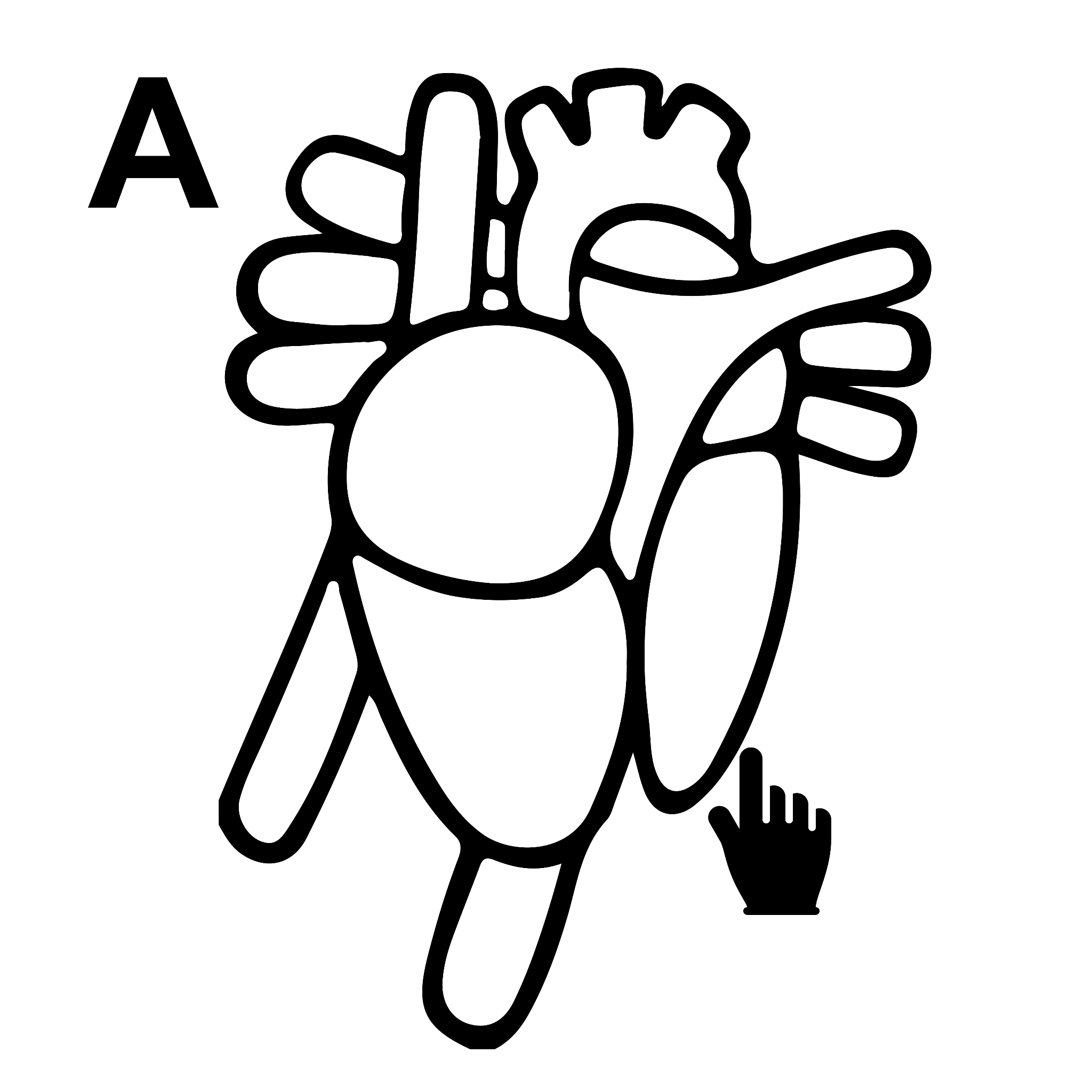

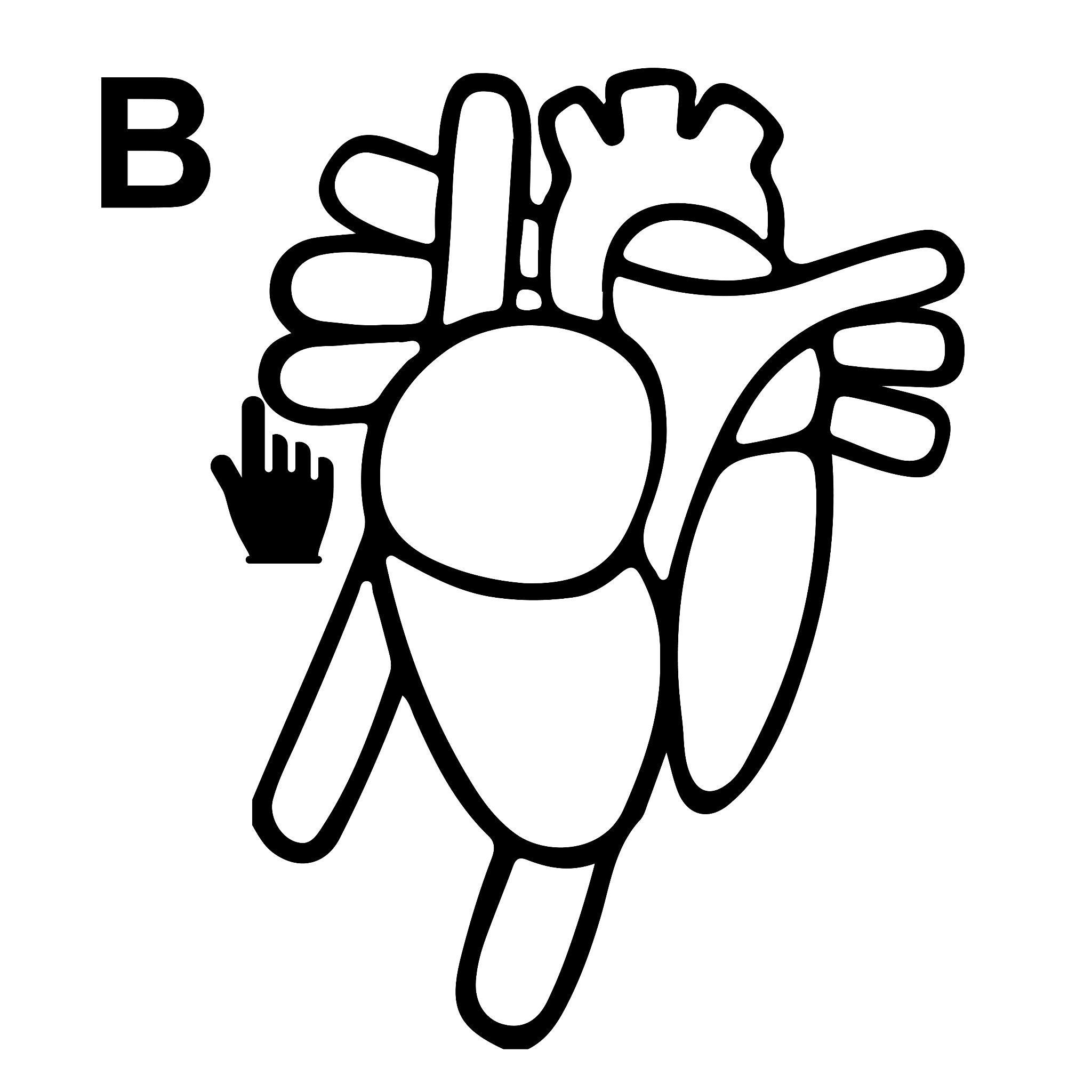
**
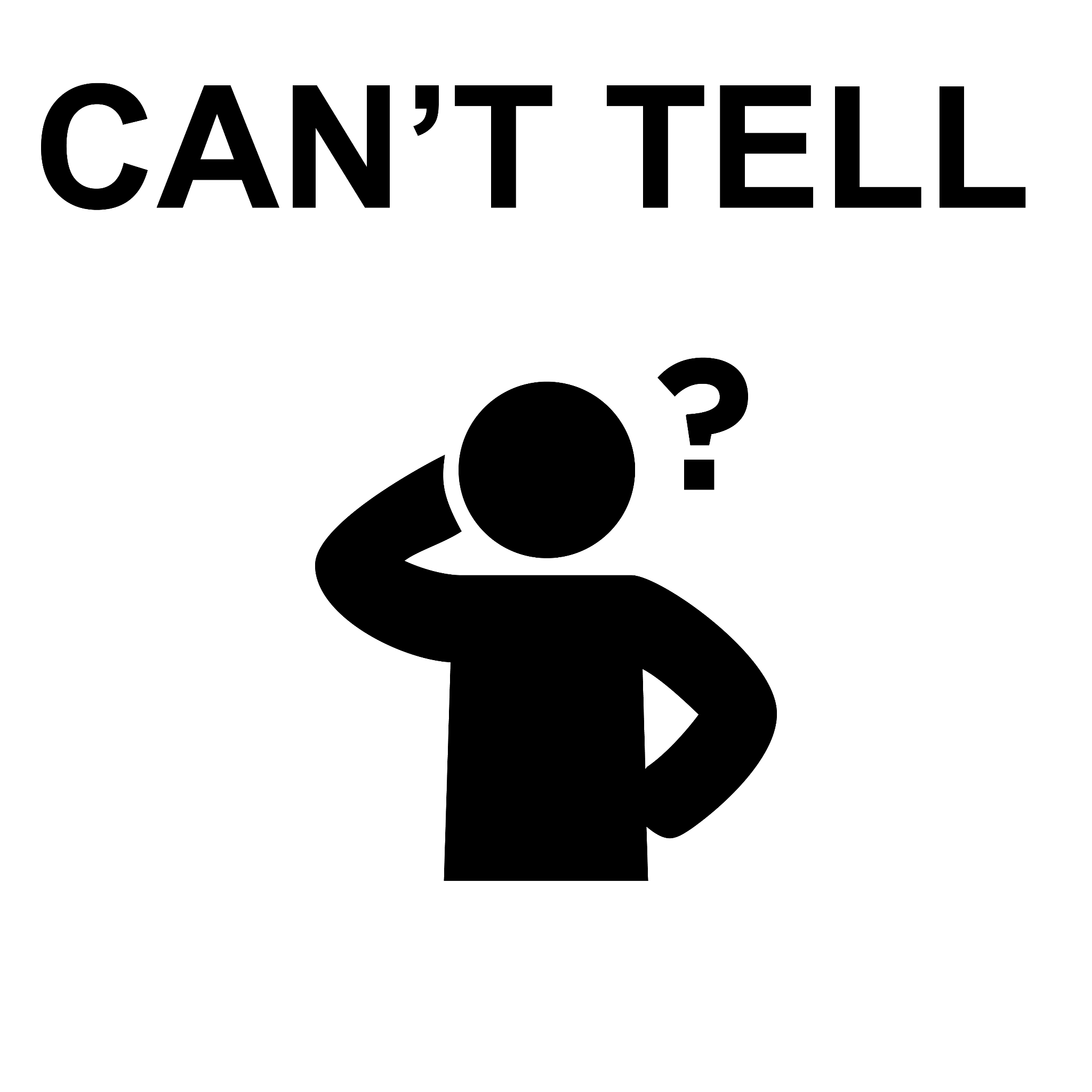
**

1. Which one do you think is better for you to do?

Point to or circle the one you think is right. It's totally okay if you're not sure!

**
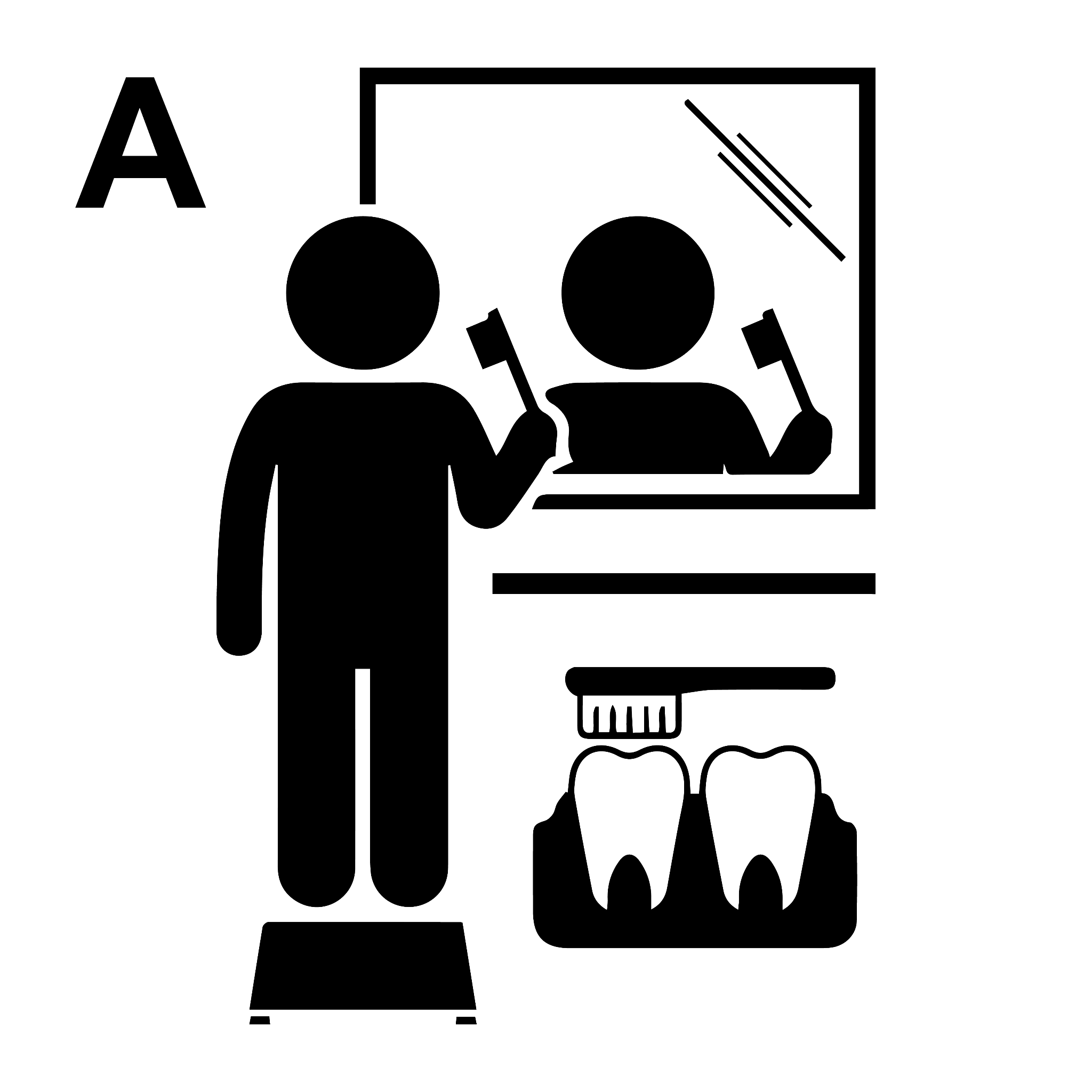

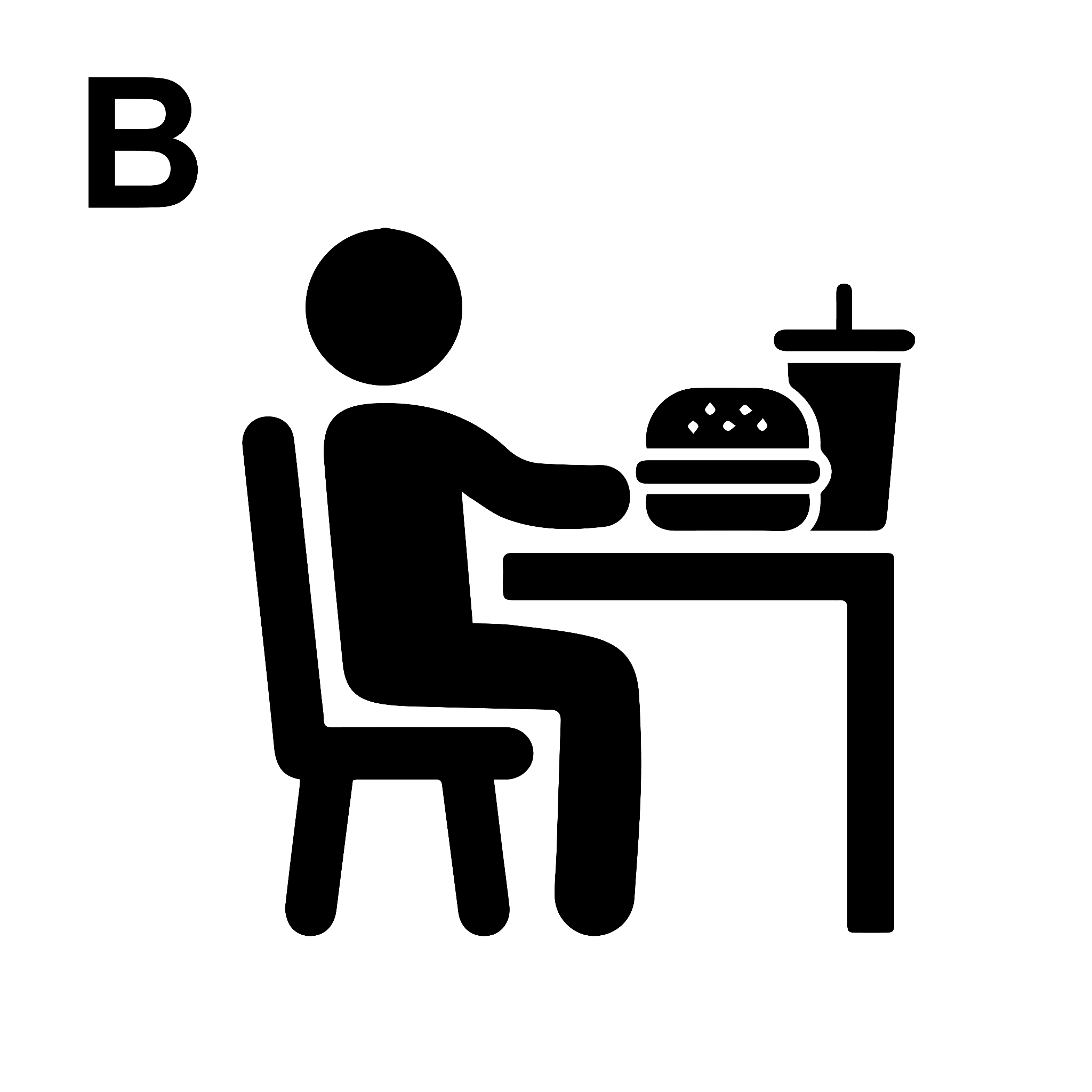

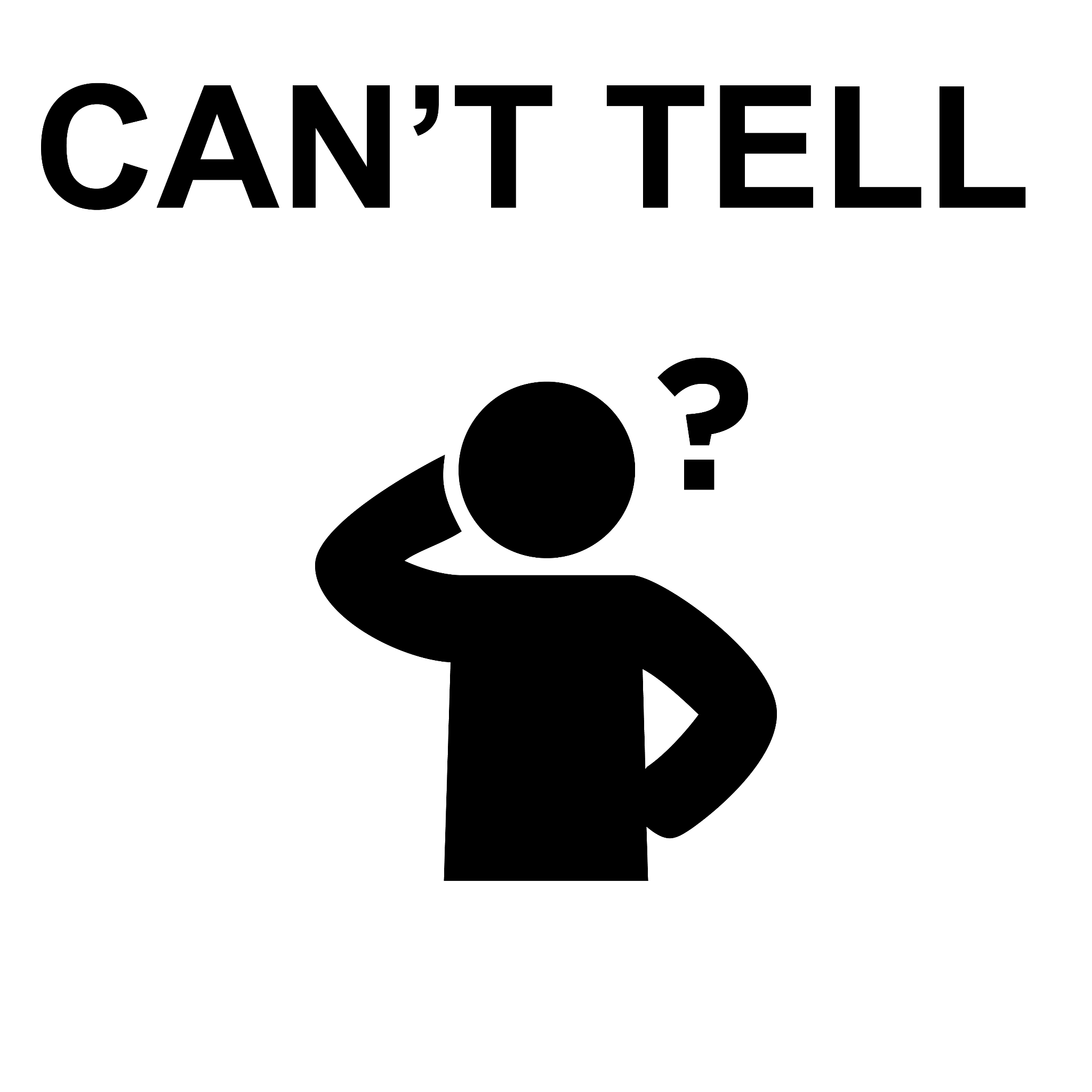
**

1. Let’s see! Which one is better for you to eat?

Point to or circle the one you think is right. It’s totally fine if you’re not sure!

**
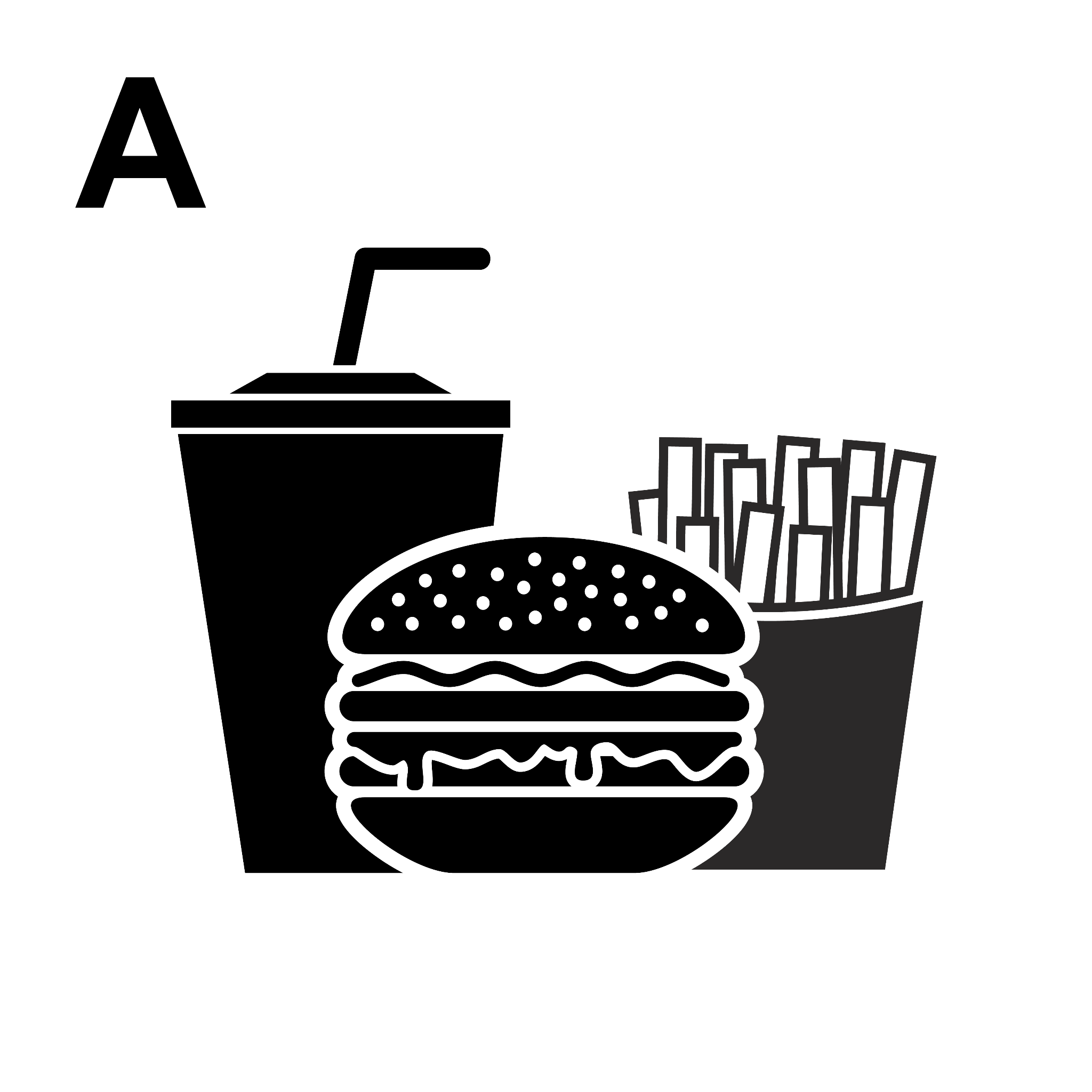

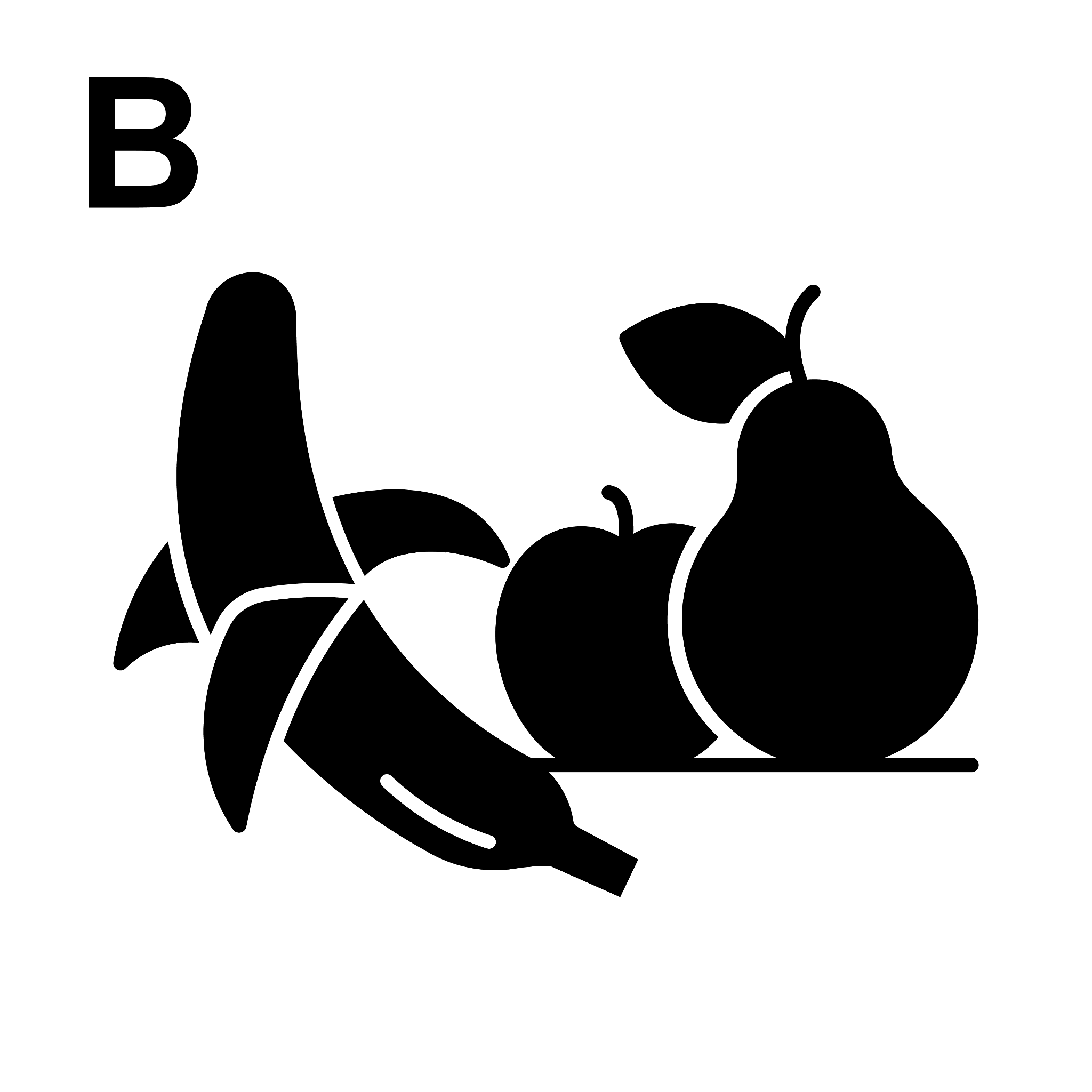

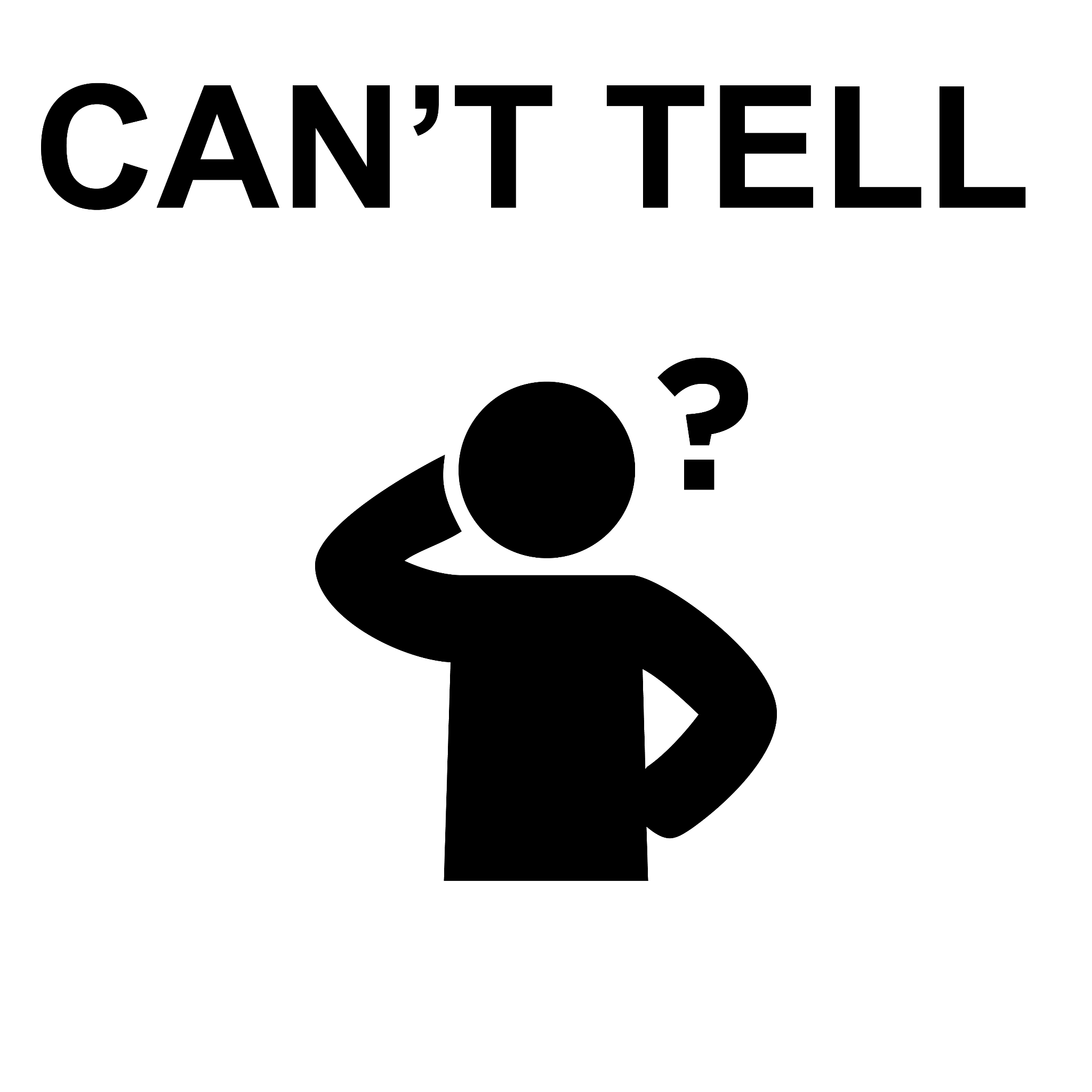
**

1. Have you ever heard of an 'EKG'? Which picture shows an EKG?

Point to or circle the one you think is right. If you’re not sure, say, “CAN’T TELL.”


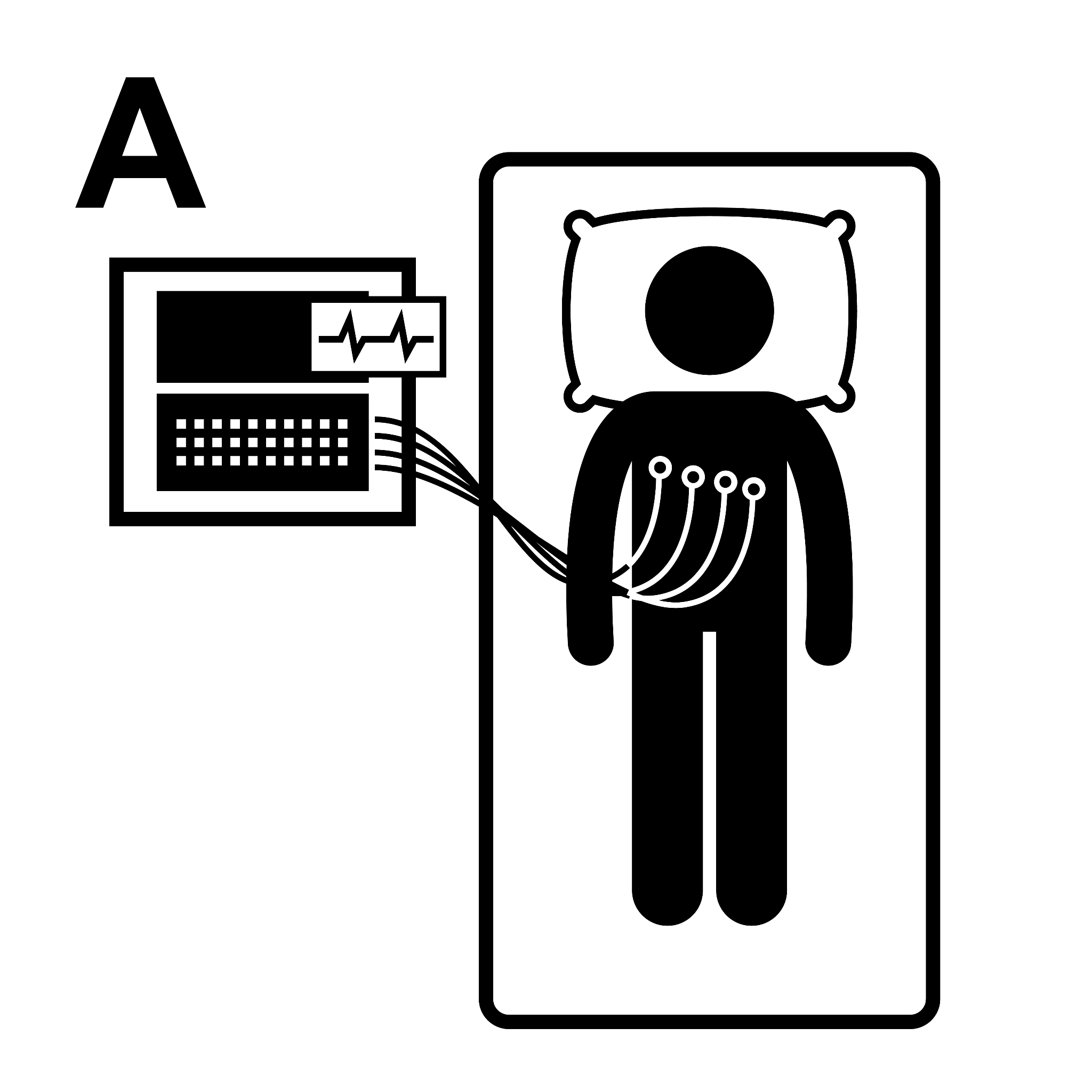

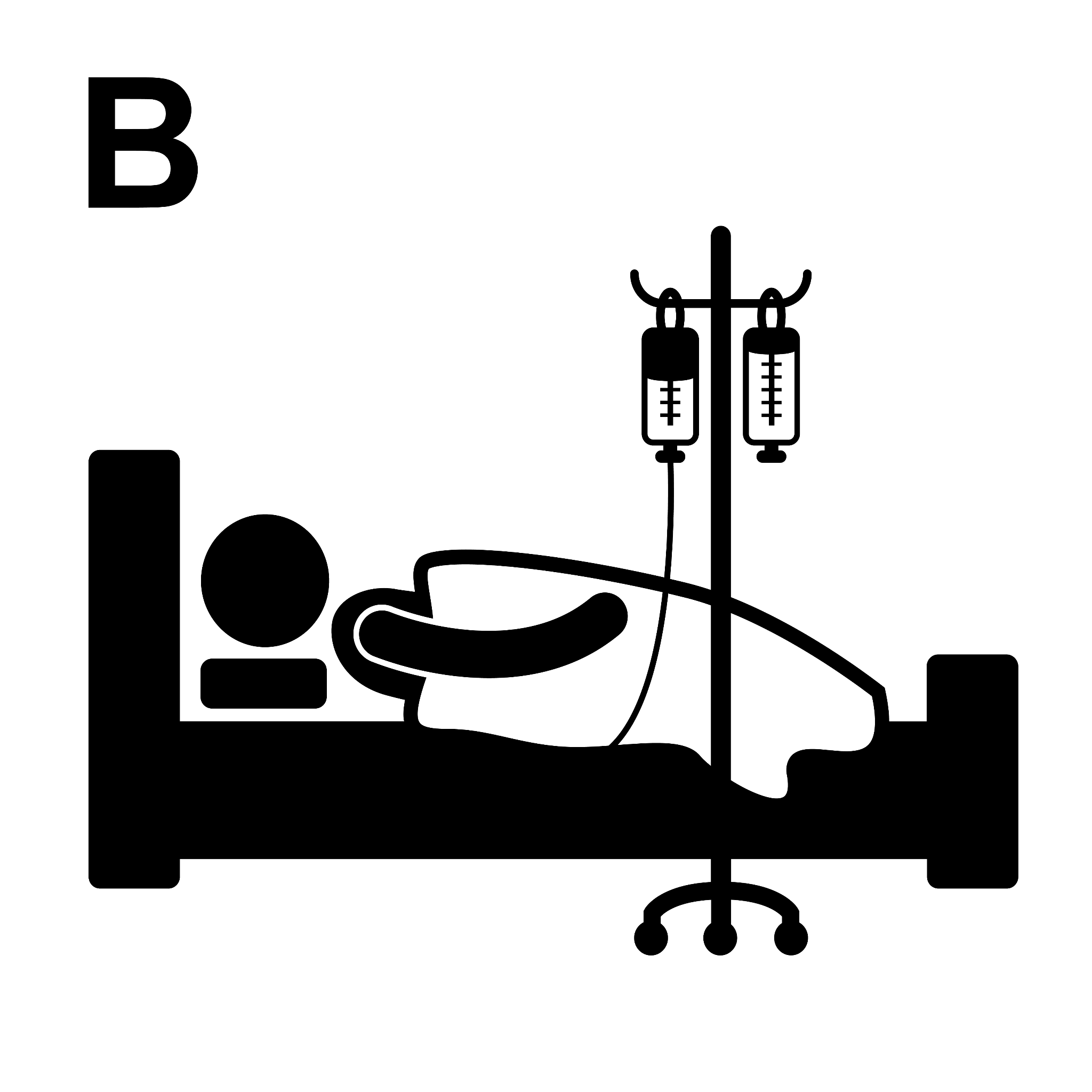
**
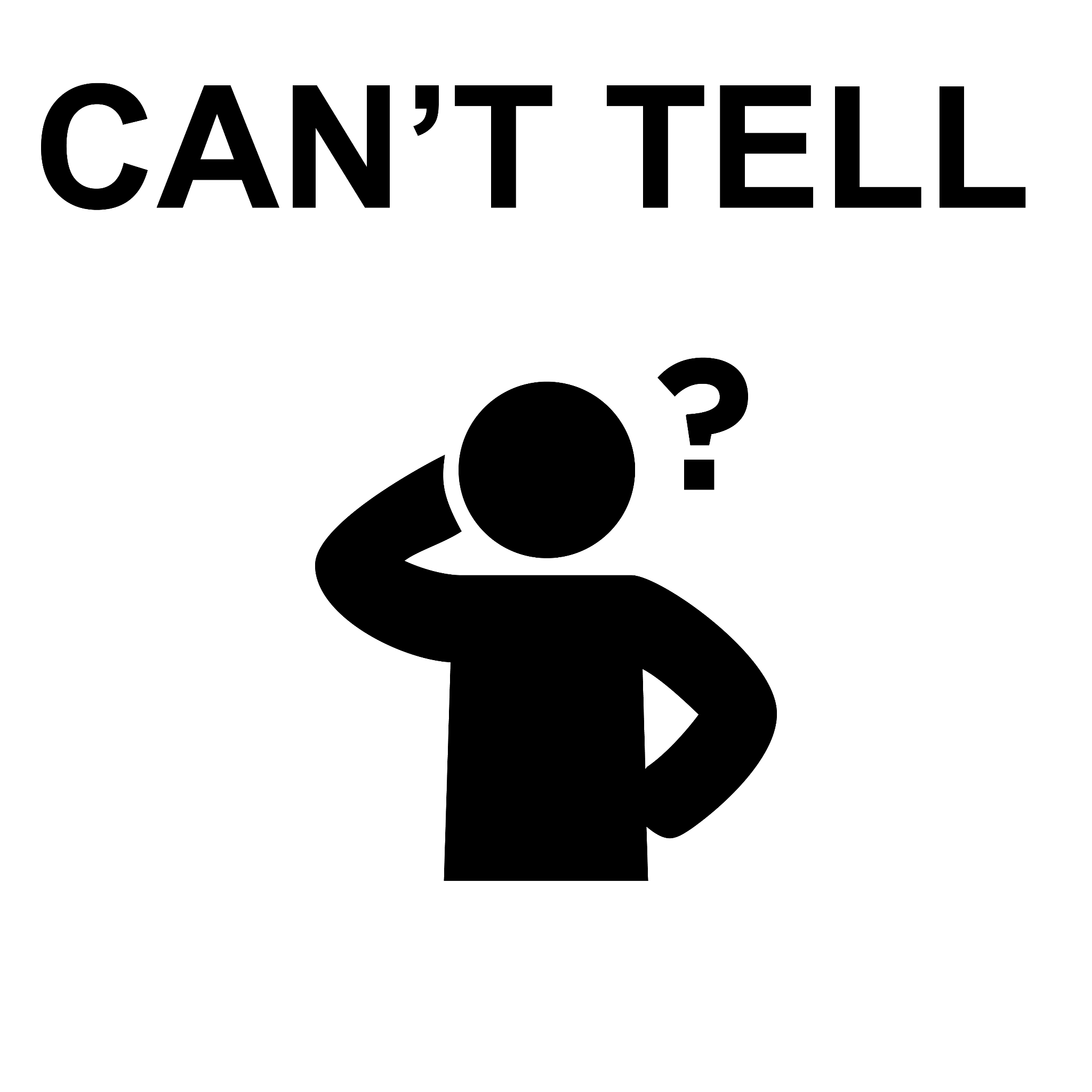
**

1. Do you know about 'Blood Pressure'? Which picture do you think shows Blood Pressure?

Point to or circle the one you think is right.

**
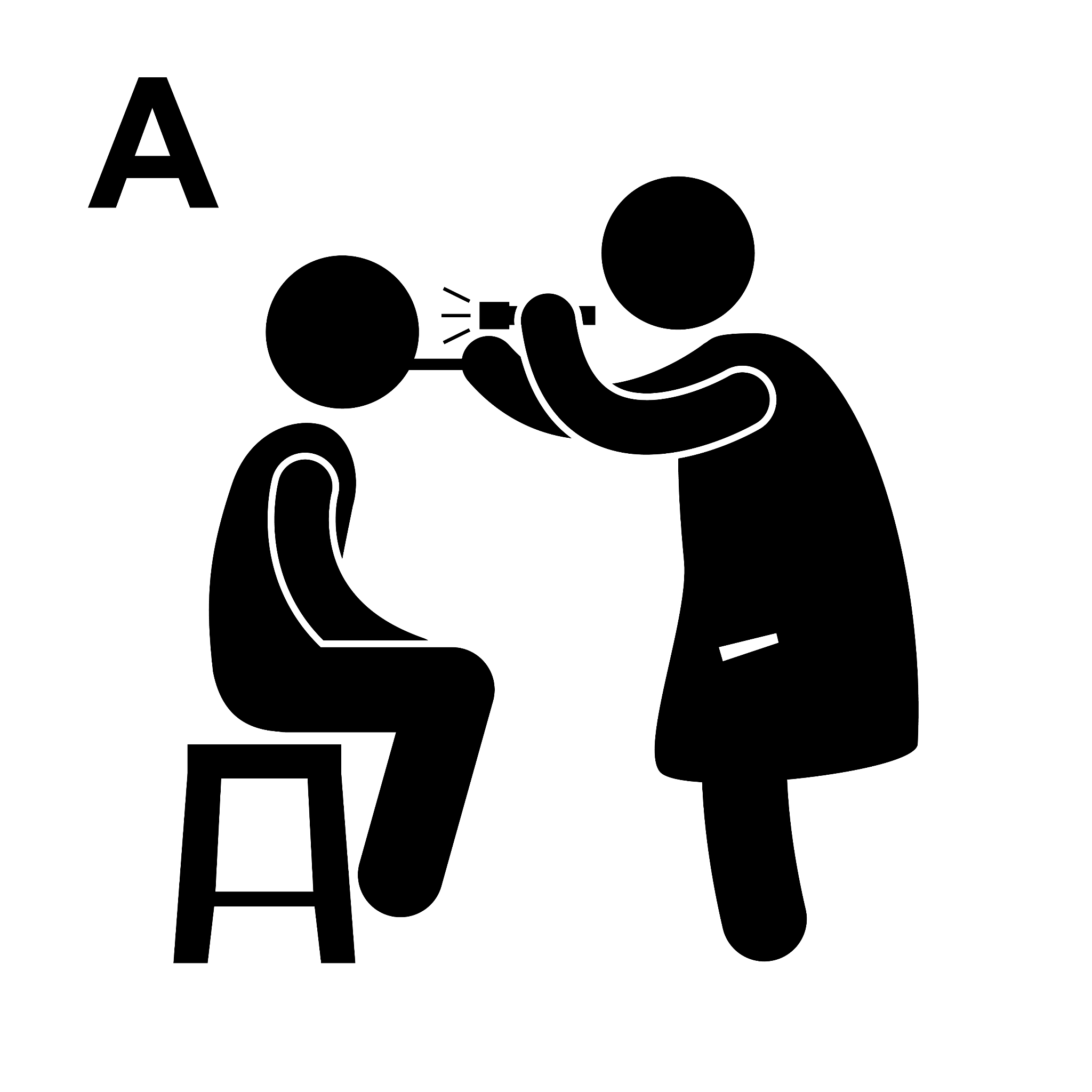

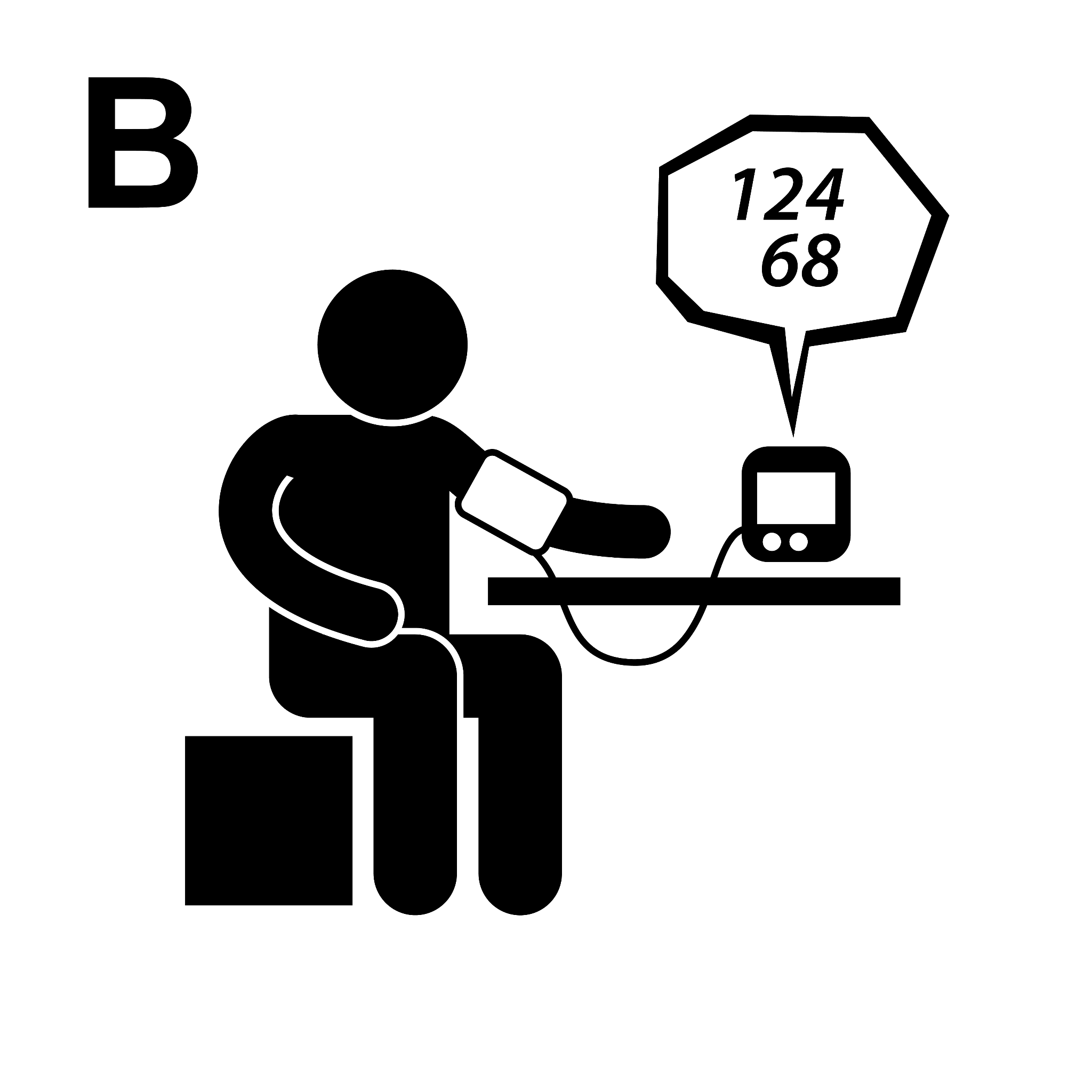

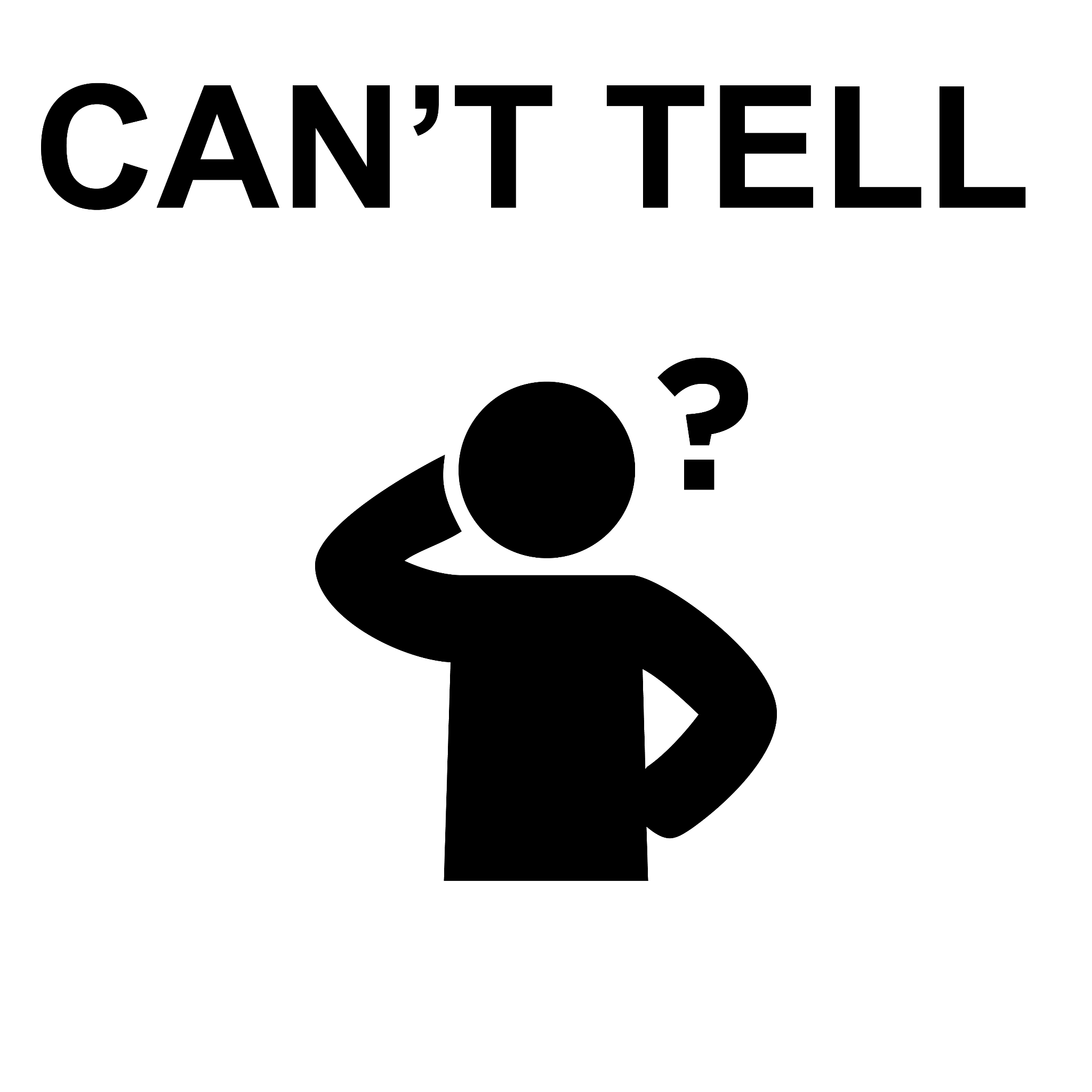
**

1. What do you usually do when you visit the doctor?

Point to or circle the picture that matches what you do. If you’re not sure, that’s perfectly okay!

**
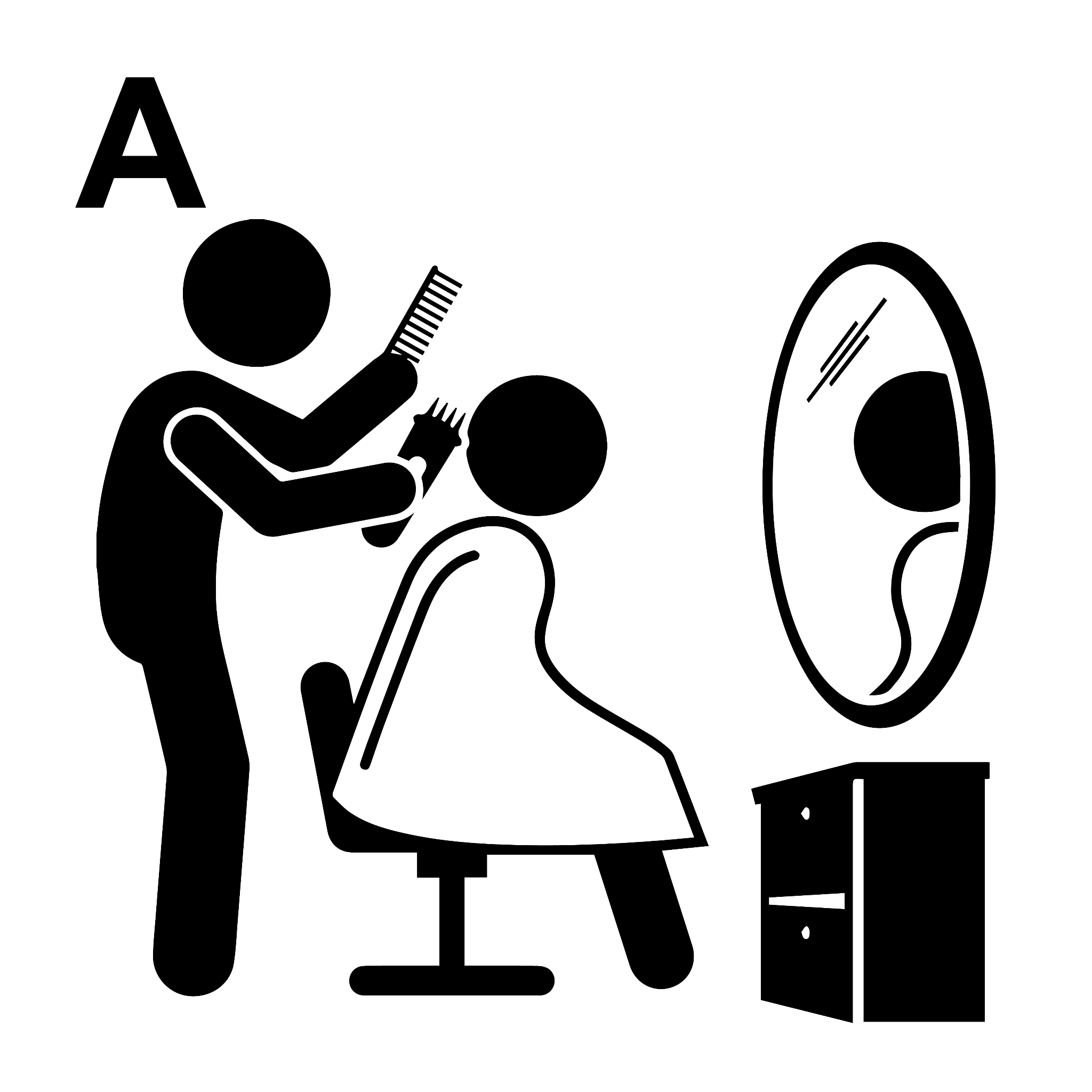

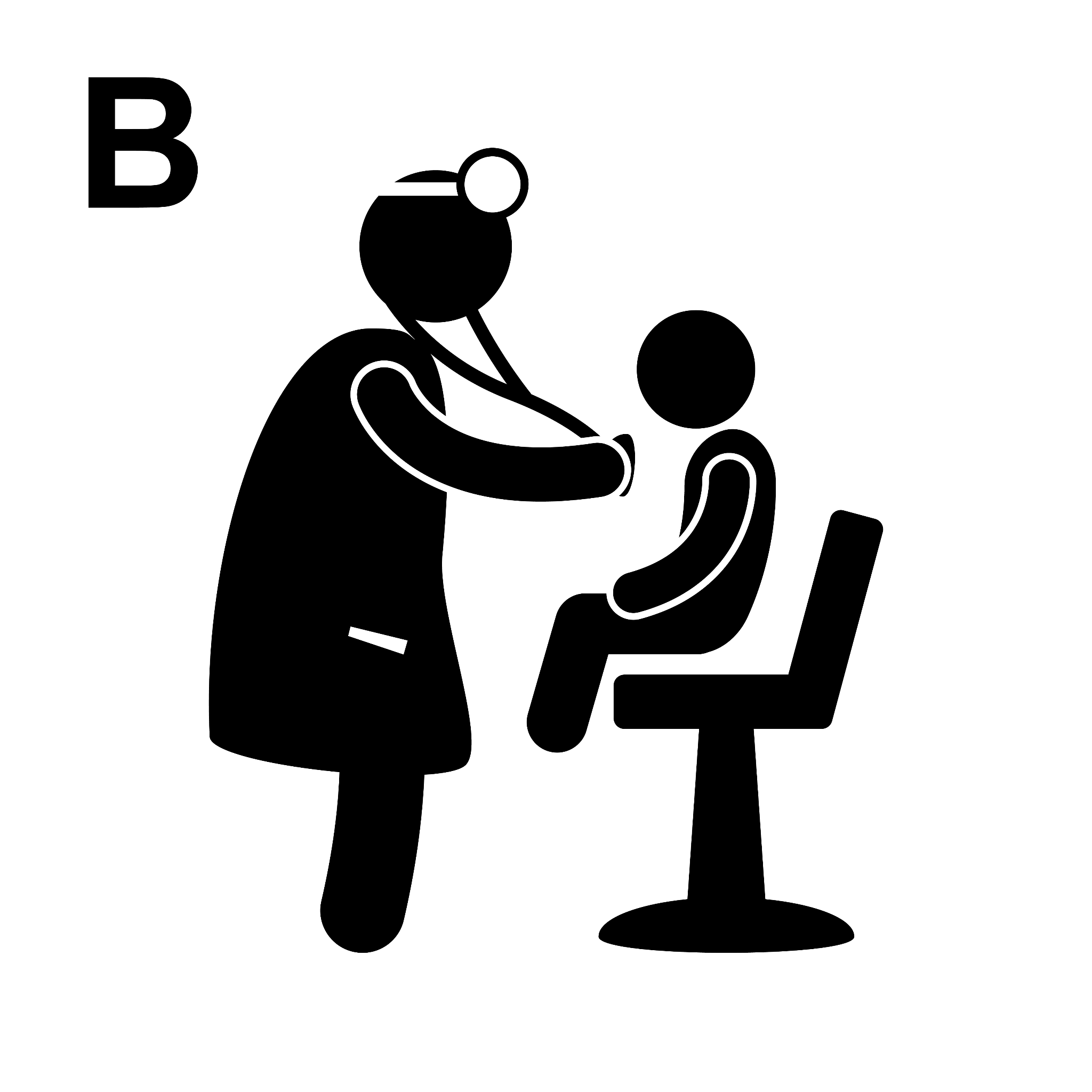

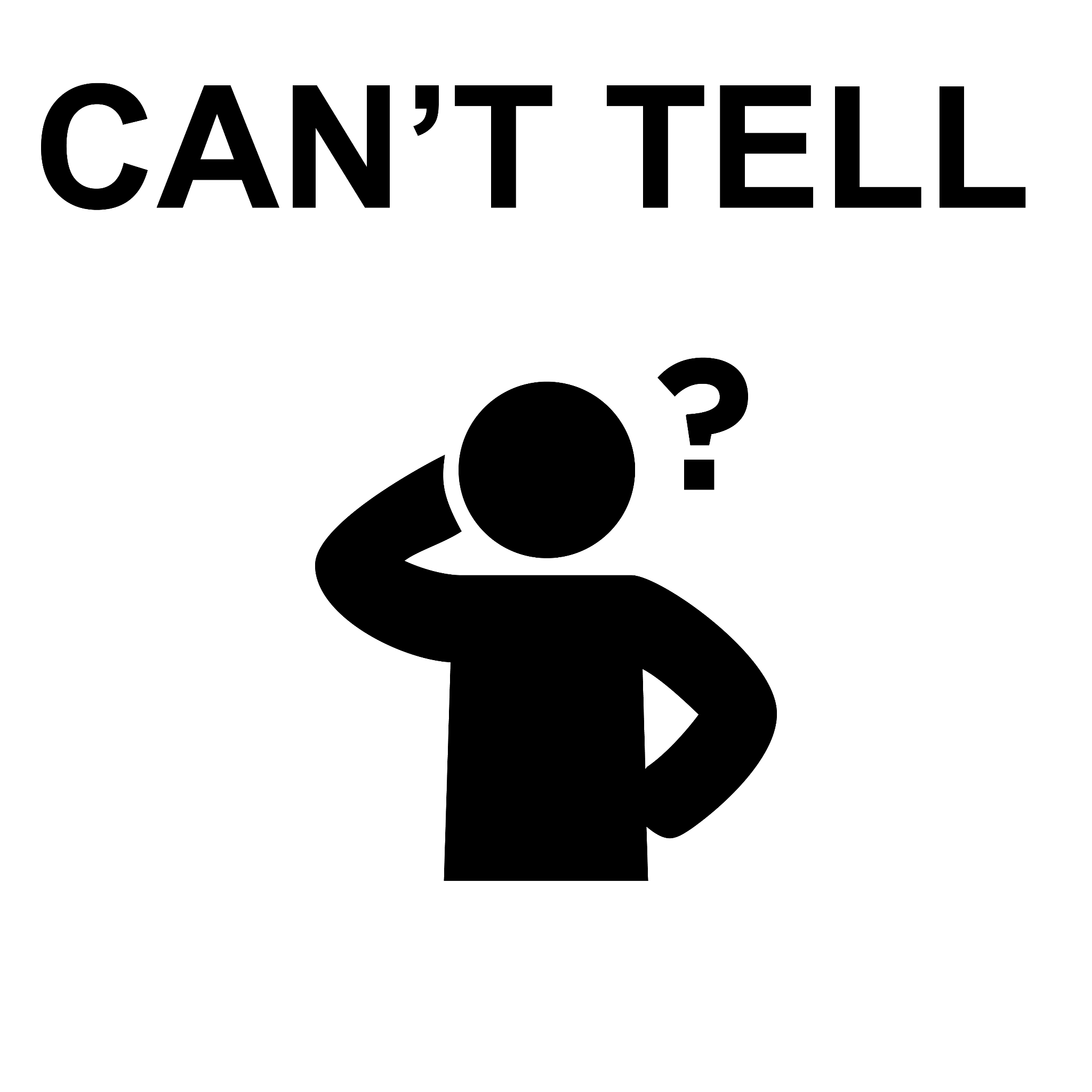
**

1. Which of these two buddies might be feeling chest pain?

Use your detective skills to point to or circle your answer. If you’re not sure, it’s okay to say, “CAN’T TELL.”


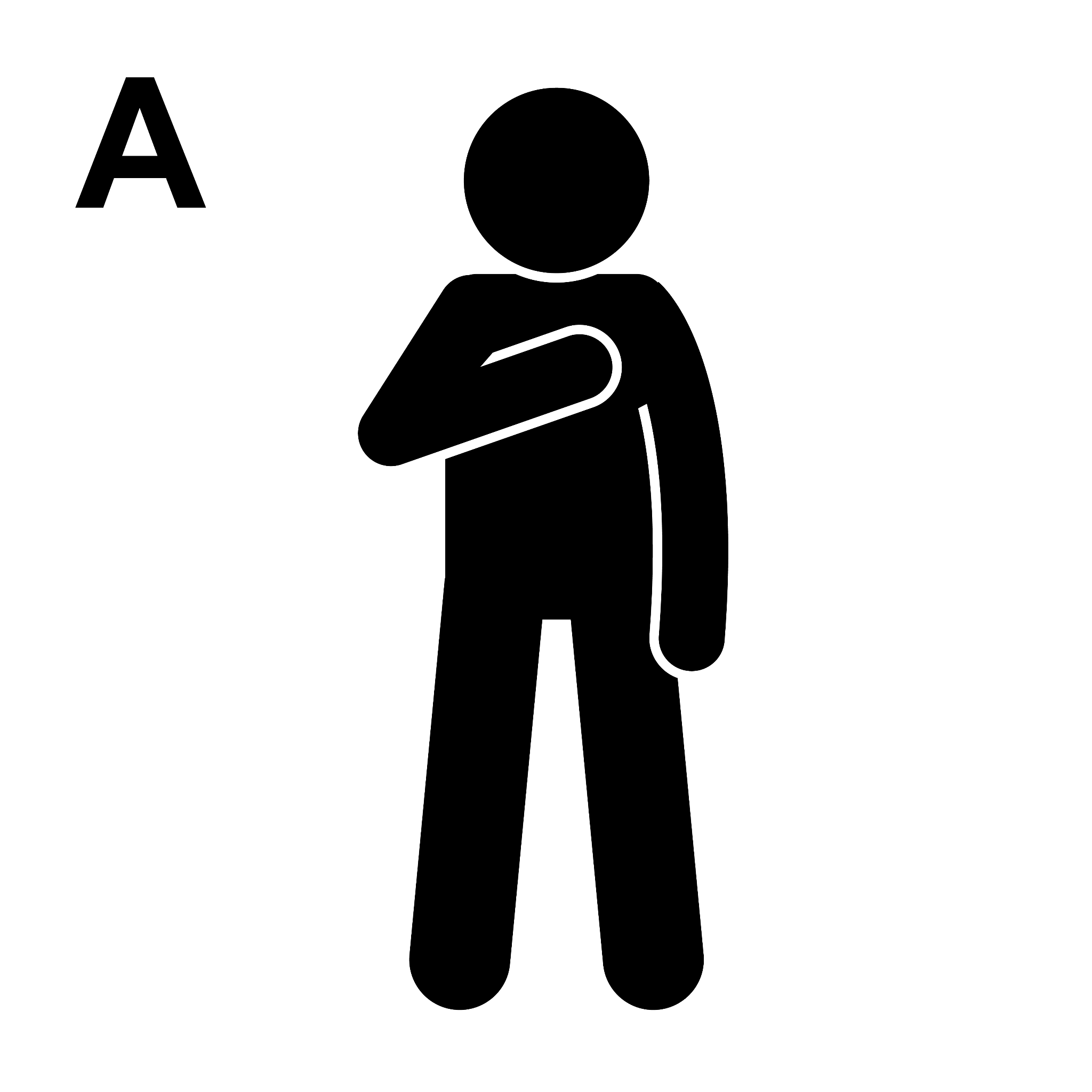
**
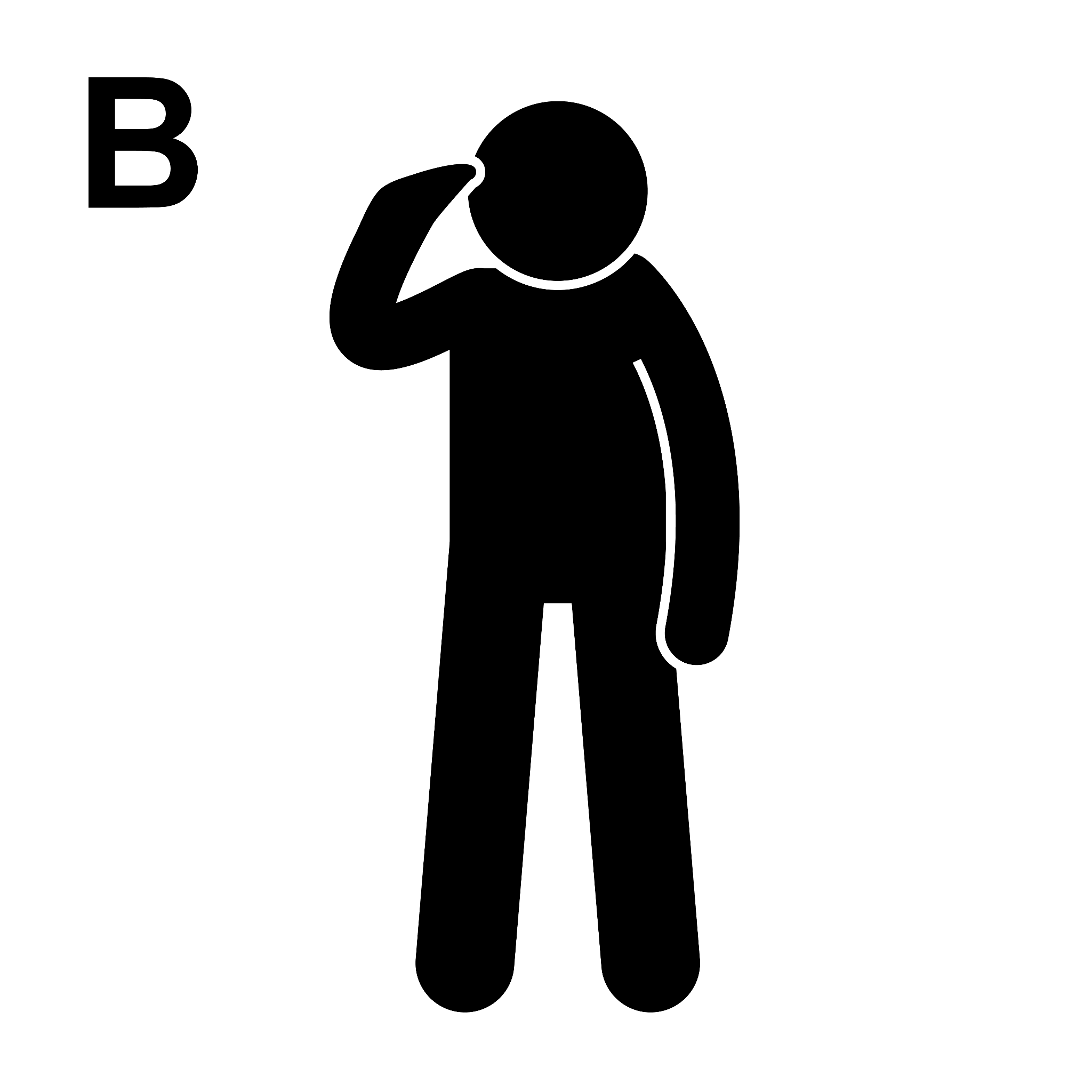

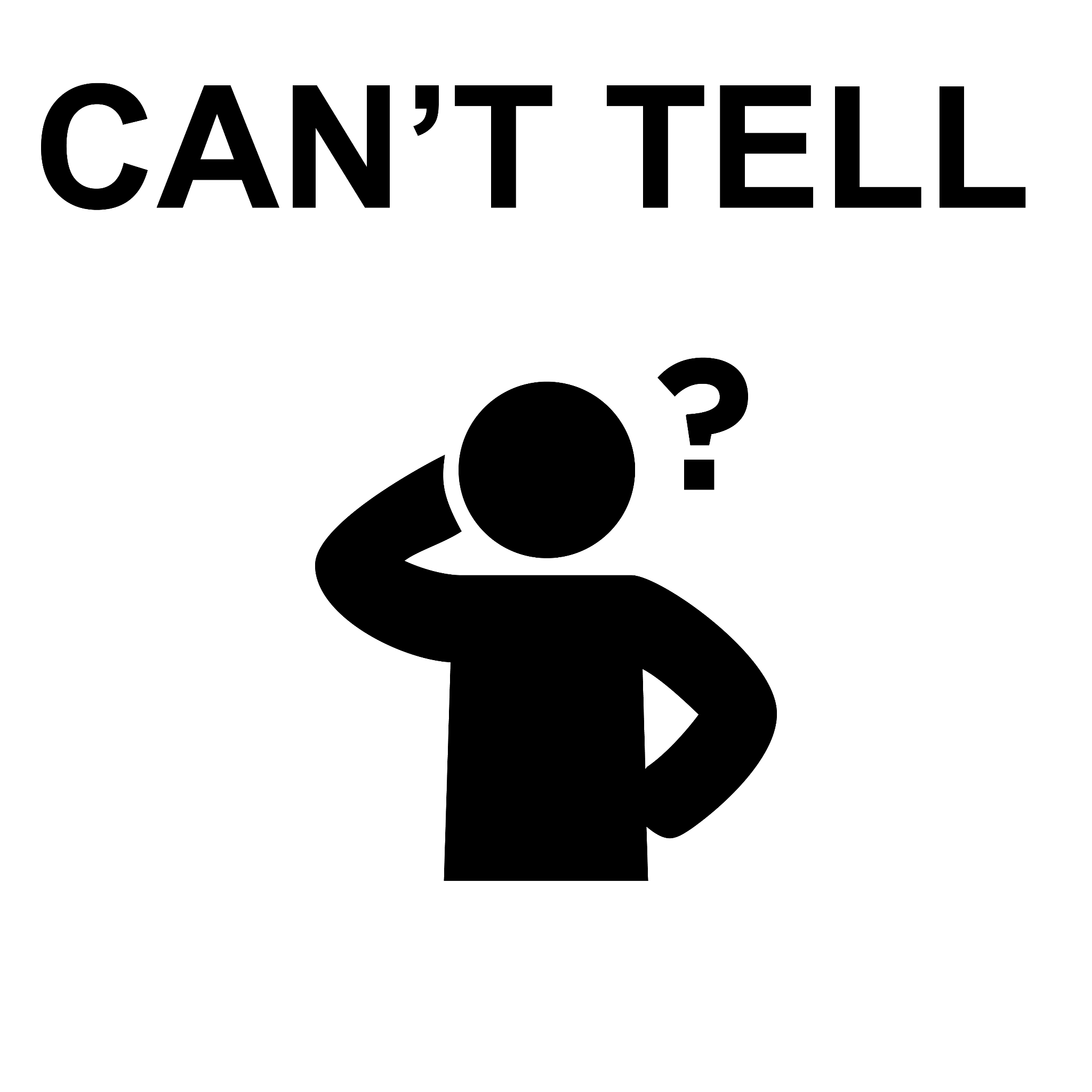
**

1. Last question detective! Which one is better for your heart?

Point to or circle the one you think is right. Thanks, Detective!


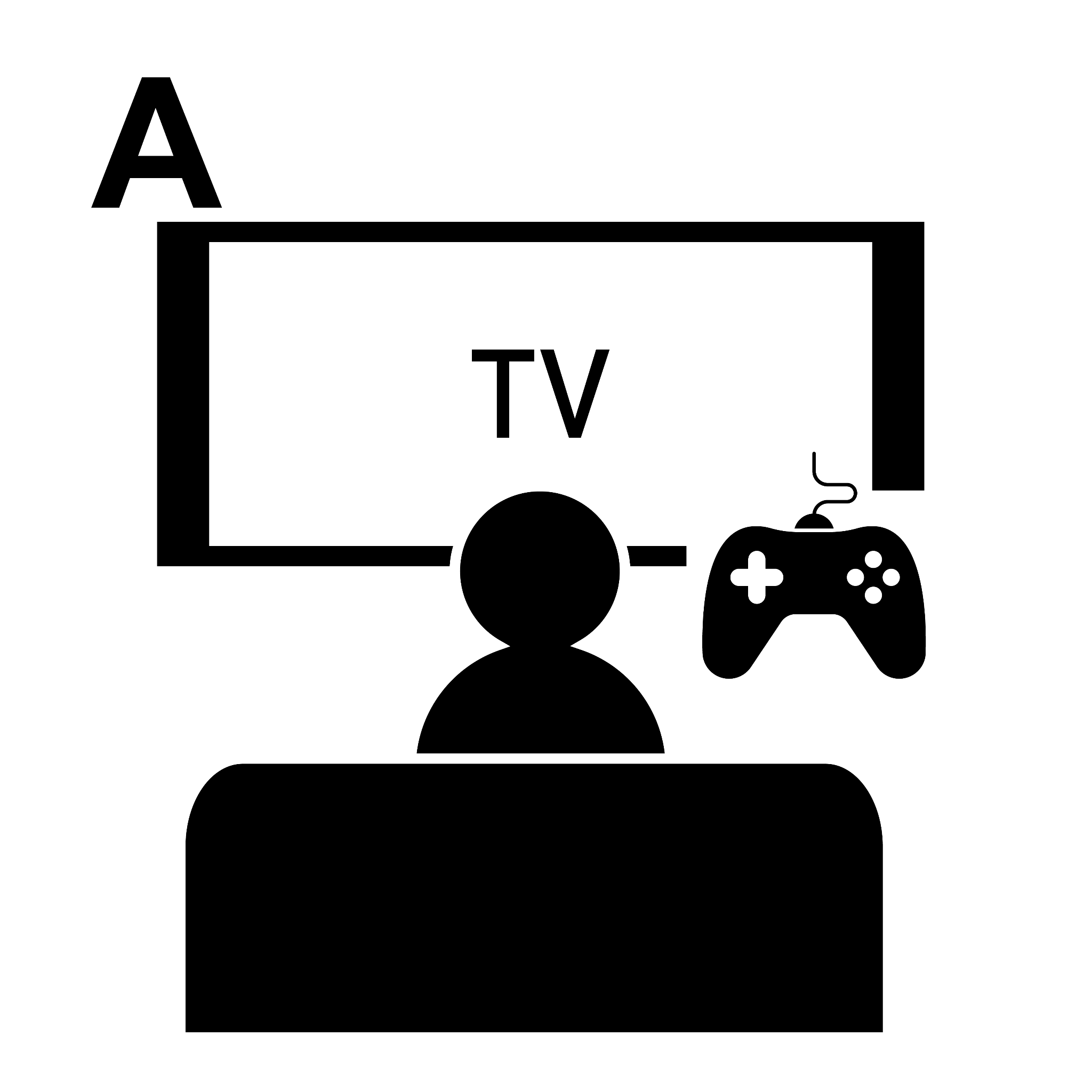
**
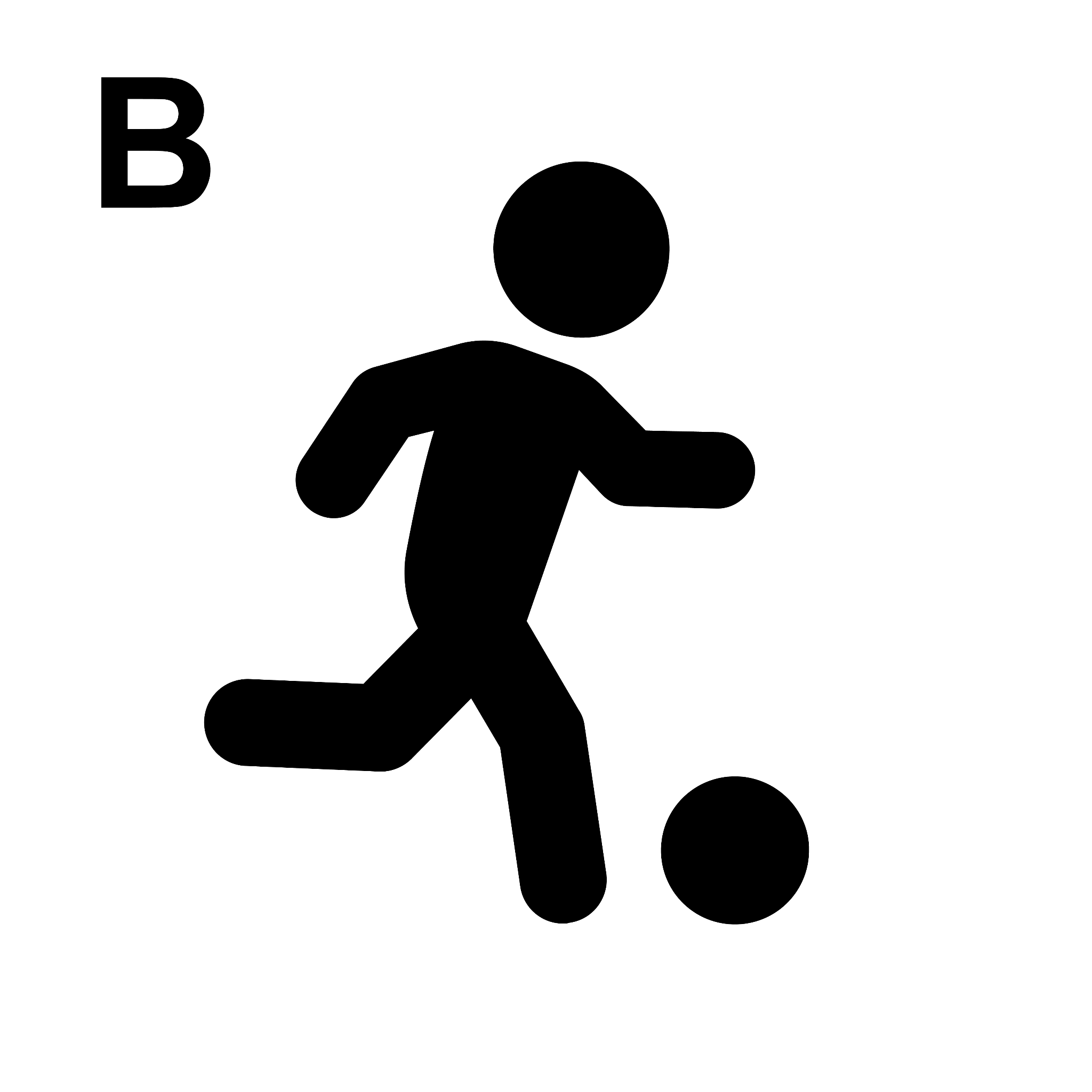

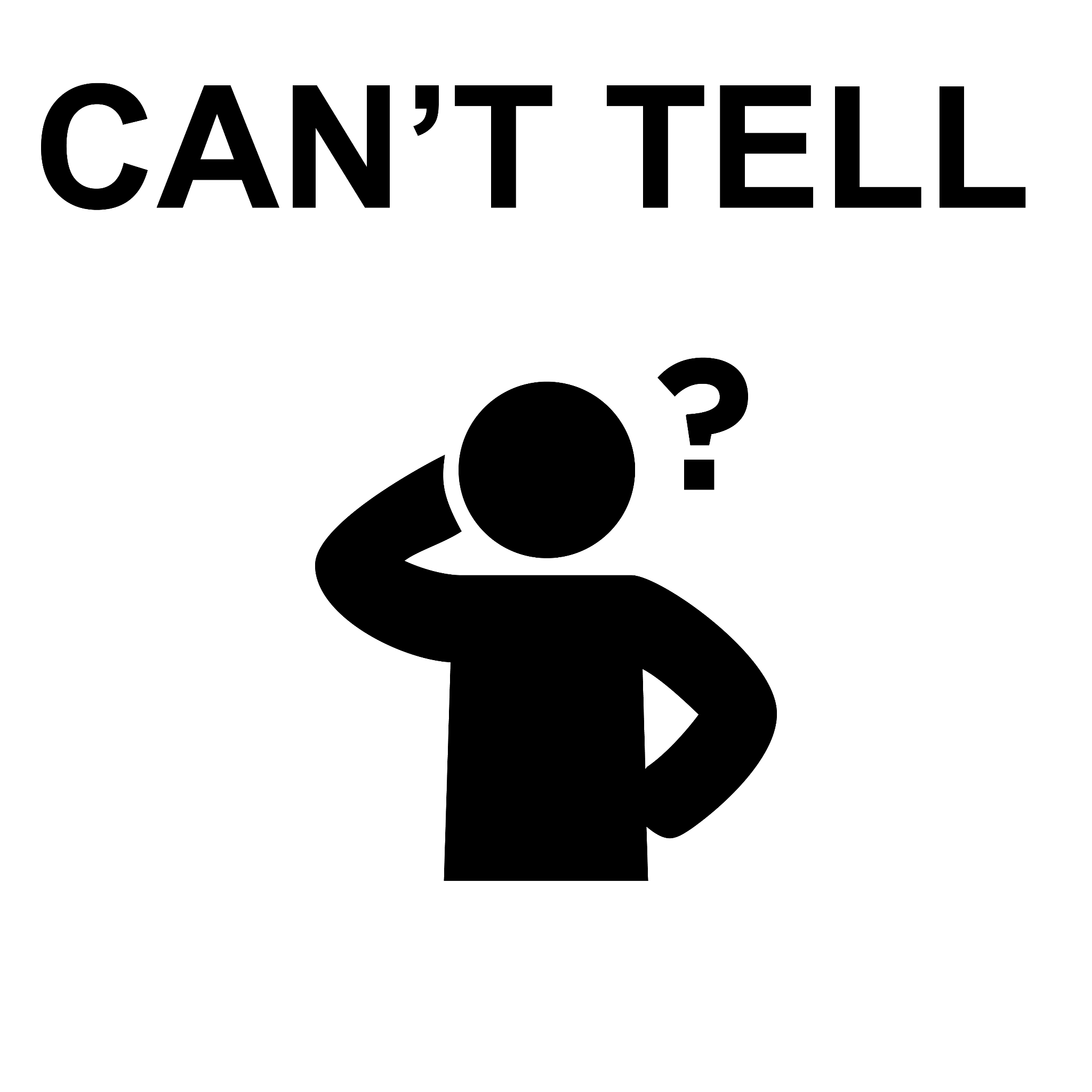
**

#### Congenital Heart Disease Parental Educational Burden Assessment (CHD-PEBA)

**ID #:**

**Month / Day / Year:**

**Pre** _____ **Post** _____

**Caregivers/Parental Burden**

**Congenital Heart Disease Education**

**Pre-Assessment - CHILDREN (4-10)**

Survey:

Thank you for participating in this survey. Your responses will remain confidential, with no right or wrong answers.

What this survey!

This survey focuses on exploring your and your children's educational needs as you navigate the challenges of congenital heart disease (CHD). It should take less than 10 minutes, and your input is greatly appreciated.

**Section 1: Basic Information**

1. Let's start simple – please select the option that best describes your **relationship** to a child with **Congenital Heart Disease (CHD)**.
   - Parent
   - Guardian
   - Other (please specify): ____________
2. And may I ask **how old** your **little one** with **CHD** is?
   - 4
   - 5
   - 6
   - 7
   - 8
   - 9
   - 10

**Section 2: Parents/Caregivers' Understanding and Experience with CHD**

*“Now, let's explore your understanding and experience in supporting your child with CHD and the challenges you may face.”*

1. How would you rate **your understanding** of the **heart's function**?

For example, if you understand the heart's basic structure and function, including its role in circulating blood throughout the body.

- - 1 – Not at all familiar
  - 2 – Slightly familiar
  - 3 – Somewhat familiar
  - 4 – Moderately familiar
  - 5 – Extremely familiar

1. How would you rate your **understanding** of your **child's Congenital Heart Disease (CHD)**?
   - 1 – Not at all familiar
   - 2 – Slightly familiar
   - 3 – Somewhat familiar
   - 4 – Moderately familiar
   - 5 – Extremely familiar
2. How **confident** are you in providing accurate **educational support** for **your child** with CHD?
   - 1 – Not at all confident
   - 2 – Slightly confident
   - 3 – Moderately confident
   - 4 – Very confident
   - 5 – Extremely confident
3. How would you rate your **stress level** related to **educating** your **child** about CHD?
   - 1 – Very low stress
   - 2 – Low stress
   - 3 – Moderate stress
   - 4 – High stress
   - 5 – Very high stress
4. How often do you consider your **child's CHD** to be a **major stressor** in your life?
   - Daily
   - Weekly
   - Monthly
   - A few times a year
   - Almost never
   - Other (please specify): ______
5. How do you usually **find information** about your **child's heart condition**?

Choose as many as you like

- - From doctors, nurses, child life specialists, or medical staff/healthcare providers during medical appointments.
  - From other parents or caregivers
  - From educational materials provided by healthcare providers.
  - From online resources (e.g., health-related websites).
  - From social media (e.g., Instagram, Facebook, TikTok).
  - From support groups or community events for CHD families.
  - From educational apps for children with CHD.
  - Other (please specify): ______

1. On average, how many **hours per week** do you spend **educating** your child about **their CHD**?

- None
- Less than 1 hour
- 1-2 hours
- 3-4 hours
- 5-6 hours
- More than 6 hours
- Other (please specify): ______

1. How **challenging** is it for you to care for your child's Congenital Heart Disease?

(If no challenge, choose "not at all challenging")

- - Balancing your child's health needs and parenting tasks - [ ] Not at all Challenging [ ] Slightly Challenging [ ] Moderately Challenging [ ] Very Challenging [ ] Extremely Challenging
  - Maintaining your own health and well-being - [ ] Not at all Challenging [ ] Slightly Challenging [ ] Moderately Challenging [ ] Very Challenging [ ] Extremely Challenging
  - Communicating with healthcare providers - [ ] Not at all Challenging [ ] Slightly Challenging [ ] Moderately Challenging [ ] Very Challenging [ ] Extremely Challenging
  - Time and effort spent educating your child about CHD - [ ] Not at all Challenging [ ] Slightly Challenging [ ] Moderately Challenging [ ] Very Challenging [ ] Extremely Challenging
  - Finding educational resources about CHD - [ ] Not at all Challenging [ ] Slightly Challenging [ ] Moderately Challenging [ ] Very Challenging [ ] Extremely Challenging

1. How do you typically **cope** with the stress and **workload** of **educating** your child about CHD?

Choose as many as you like

- Seeking support from a healthcare provider.
- Joining support groups for parents of children with CHD.
- Utilizing online resources and educational materials.
- Engaging in self-care activities (e.g., exercise, meditation).
- Relying on family and friends for assistance.
- I have no stress.
- Other (please specify): ______

**Section 3: Child's Understanding and Experience**

*"Next, we’ll evaluate your child's understanding of their condition and their comfort level with different healthcare experiences related to CHD."*

1. How would you **rate** your **child's understanding of CHD**?
   - 1 – Not at all familiar
   - 2 – Slightly familiar
   - 3 – Somewhat familiar
   - 4 – Moderately familiar
   - 5 – Extremely familiar
2. And how does your **child** typically **learn** about their condition?

Choose as many as you like

- - From doctors, nurses, child life specialists, or medical staff/healthcare providers during medical appointments.
  - From educational materials provided by healthcare providers.
  - From support groups or community events for CHD families.
  - From information shared by myself (parents or caregivers).
  - From other CHD children.
  - From educational apps for children with CHD.
  - From educational toys.
  - Other (please specify): ______

1. How would you **rate** your child's **comfort level** with the following **healthcare experiences**:
   - Follow-up visits - [ ] Very Uncomfortable [ ] Uncomfortable [ ] Neutral [ ] Comfortable [ ] Very Comfortable
   - Interacting with healthcare staff - [ ] Very Uncomfortable [ ] Uncomfortable [ ] Neutral [ ] Comfortable [ ] Very Comfortable
   - Medical procedures (like blood tests, EKG) - [ ] Very Uncomfortable [ ] Uncomfortable [ ] Neutral [ ] Comfortable [ ] Very Comfortable
   - Surgery - [ ] Very Uncomfortable [ ] Uncomfortable [ ] Neutral [ ] Comfortable [ ] Very Comfortable
   - Overall healthcare experience - [ ] Very Uncomfortable [ ] Uncomfortable [ ] Neutral [ ] Comfortable [ ] Very Comfortable

**Section 4: Educational Support and Materials**

*“In this section, we'll explore the support available for educating your child about CHD and assess the effectiveness of these resources.”*

1. How likely do you think you need **help** to ensure that the information you provide to your child about their CHD is **correct**?
   - 1 – Not likely at all
   - 2 – Unlikely
   - 3 – Neutral
   - 4 – Likely
   - 5 – Highly likely
2. Do you receive any **educational materials** during your child's doctor visits?
   - Yes
   - No
3. IF yes, How would you **rate** the **educational** materials provided by healthcare providers for your child's health based on the following **specifications**?
   - Easy to understand - [ ] Strongly Disagree [ ] Disagree [ ] Neutral [ ] Agree [ ] Strongly Agree
   - Suitable for your child's age (age-appropriate) - [ ] Strongly Disagree [ ] Disagree [ ] Neutral [ ] Agree [ ] Strongly Agree
   - Engaging - [ ] Strongly Disagree [ ] Disagree [ ] Neutral [ ] Agree [ ] Strongly Agree
   - Overwhelming - [ ] Strongly Disagree [ ] Disagree [ ] Neutral [ ] Agree [ ] Strongly Agree
   - Effective - [ ] Strongly Disagree [ ] Disagree [ ] Neutral [ ] Agree [ ] Strongly Agree

**Section 5: Parents Demographic Information**

*“Thank you. We're almost done; let's know more about you!”*

1. Please let us know how you **prefer** to be **identified**.
   - Female
   - Male
   - Non-binary
   - Prefer not to say
2. And what is **your age** **range**?
   - Under 25
   - 25 - 34
   - 35 - 44
   - 45 - 54
   - 55 or older
   - Prefer not to say
3. Which **race or ethnicity** best describes you?
   - White / Caucasian
   - African American / Black
   - Hispanic / Latino
   - Asian / Pacific Islander
   - Native American / Alaskan Native
   - Native Hawaiian / Other Pacific Islander
   - Middle Eastern / North African
   - Prefer not to say
4. And what is your **highest level of education**?
   - Less than High School
   - High School Diploma/GED
   - Some College/AA Degree/Technical School Training
   - Bachelor's Degree
   - Master's Degree
   - Doctoral Degree
   - Other (please specify): ______
5. What is your current **employment** status?
   - Employed Full-Time (40 or more hours per week)
   - Employed Short-Time (less than 40 hours per week)
   - Self-employed
   - Unemployed
   - Student
   - Retired
6. What is your annual **household** **income**?
   - Less than $25,000
   - $25,000 - $50,000
   - $50,000 - $75,000
   - $75,000 - $100,000
   - More than $100,000
   - Prefer not to say
7. And may I ask what is your **marital** status?
   - Single
   - Married
   - Divorced
   - Widowed
   - Other (please specify): ______
8. Finally, which **language** do you feel **most comfortable** speaking?

Type or select an option

**Section6: Additional Comments and Contact Information**

1. Thanks a lot. Now, is there **anything else** you'd like to tell us about the **challenges** and **workload** you experience as a **parent** of a child with CHD?

Type your answer here:

1. Before we go, can we get your **full name**?

Please provide your **full name** so we can send you **incentives**.

Your **information** will be kept **confidential** and used solely for survey communication.

1. And, what **email address** can we reach you at?

This is only to confirm we received your answers and sent your **incentive**.

**Thank you so much for your time and feedback!**

For any questions or feedback, please don't hesitate to contact us.

#### Congenital Heart Disease Healthcare Provider Educational Efficiency Assessment (CHD-HEEA)

**ID #:**

**Month / Day / Year:**

**Pre** _____ **Post** _____

**Healthcare Providers' Efficiency**

**(Workflow Optimization)**

**Congenital Heart Disease Education**

**Pre-Assessment - CHILDREN (4-10)**

Survey:

Thank you for participating in this survey. Your input is invaluable in our mission to support families dealing with Congenital Heart Disease (CHD) and enhance care delivery.

About the Survey:

This survey aims to understand the **time and effort** spent **educating** children and parents during visits to improve your workflow and care coordination. It should take 5 to 10 minutes to complete, and we would greatly appreciate your input.

**Section 1: Demographic Information**

1. **How long** have you been a healthcare provider specializing in pediatric cardiology or CHD care?
   - Less than 1 year
   - 1-5 years
   - 6-10 years
   - More than 10 years
   - Prefer not to say
2. What is your current **role** in the healthcare provider team?
   - Pediatric Cardiologist
   - Pediatric Cardiology Fellow
   - Pediatric Cardiology Nurse
   - Child Life Specialist
   - Other (please specify): ____________
3. Please specify your gender identity:
   - Male
   - Female
   - Non-binary
   - Prefer not to say
4. And what is your age range?
   - Under 25
   - 25-34
   - 35-44
   - 45-54
   - 55 or older
   - Prefer not to say

**Section 2: Educational Materials and Strategies**

1. What **educational materials/strategies** do you currently use to **educate parents/caregivers** and **CHD children** about the condition?

Choose as many as you like

- - Pre-appointment Educational Materials
  - Use of Visual Aids and Models
  - Pamphlet, Booklet, and Brochure for Parents/Caregivers
  - Educational Toys/Games (designed for education, not any toy)
  - Encouraging Questions from Parents/Caregivers
  - After Visit Summary (AVS)
  - Providing Digital Resources for Self-education
  - Other (please specify): ______

1. Please **select** the option that best describes the **educational** materials you provide to **children** with CHD and their **parents/caregivers**.
   - Easy to understand - [ ] Not at all [ ] Slightly [ ] Moderately [ ] Very [ ] Extremely
   - Suitable for CHD children's age - [ ] Not at all [ ] Slightly [ ] Moderately [ ] Very [ ] Extremely
   - Engaging - [ ] Not at all [ ] Slightly [ ] Moderately [ ] Very [ ] Extremely
   - Overwhelming - [ ] Not at all [ ] Slightly [ ] Moderately [ ] Very [ ] Extremely
   - Effective - [ ] Not at all [ ] Slightly [ ] Moderately [ ] Very [ ] Extremely
   - Likelihood of future reuse or recall - [ ] Not at all [ ] Slightly [ ] Moderately [ ] Very [ ] Extremely
2. On a scale from **'1' (Very dissatisfied)** to **'5' (Very satisfied)**, please indicate how satisfied you are with the current **educational methods** used in your practice for CHD children and their parents.
   - 1 - Very dissatisfied
   - 2 - Dissatisfied
   - 3 - Neutral
   - 4 - Satisfied
   - 5 - Very satisfied

**Section 3: Time and Effort in Education**

1. On average, how much **time** do you spend going through the **educational component** of the visit?
   - New Patient - [ ] Less than 15 min [ ] 15-30 min [ ] 30-45 min [ ] More than 1 hr
   - Follow-up Patient - [ ] Less than 15 min [ ] 15-30 min [ ] 30-45 min [ ] More than 1 hr
2. Please **rate** the **frequency** and the **effort/time** spent **educating** CHD **children** and their **parents/caregivers** about their condition in your current practice.

(1) Low - (2) Moderate - (3) Average - (4) High - (5) Very High

- - Frequency of educating **(new patient)** - [1] Low [2] Moderate [3] Average [4] High [5] Very High
  - Frequency of educating **(follow-up patient)** - [1] Low [2] Moderate [3] Average [4] High [5] Very High
  - Effort/time to teach **(new patient)** - [1] Low [2] Moderate [3] Average [4] High [5] Very High
  - Effort/time to teach **(follow-up patient)** - [1] Low [2] Moderate [3] Average [4] High [5] Very High

1. On a scale of 1 to 5, with **(1 being Not efficient at all)** and **(5 being very efficient)**, how efficient do you feel your current visits with CHD children and their parents are?"
   - 1 - Not efficient at all
   - 2 - Somewhat inefficient
   - 3 - Neutral
   - 4 - Somewhat efficient
   - 5 Very efficient

**Section 4: Challenges and Additional Resources**

1. "On a scale of 1 to 5, how much do you agree or disagree with the following statement:

“Managing **time** and **patient care** during appointments has been challenging due to the **need** for **extensive education** about CHD."

- - 1 - Strongly disagree
  - 2 - Disagree
  - 3 - Neither agree/disagree
  - 4 - Agree
  - 5 - Strongly agree

1. How **often** do you feel the need to provide **additional educational** resources to CHD children and their parents in your role?
   - Frequently
   - Occasionally
   - Rarely
   - Never
   - Other (please specify): ____________

**Section 5: Health Literacy and Communication**

1. Considering your most **vulnerable** population in terms of socioeconomic status, how **well-informed** do you think **parents/caregivers** are about their **children's condition**?
   - 1 - Not informed at all
   - 2 - Not very informed
   - 3 - Somewhat informed
   - 4 - Well-informed
   - 5 - Very well-informed
2. In your opinion, how **well-informed** are the CHD **children** themselves about their heart condition and healthcare needs?
   - 1 - Not informed at all
   - 2 - Not very informed
   - 3 - Somewhat informed
   - 4 - Well-informed
   - 5 - Very well-informed

**Section 6: Additional Comments**

1. Thanks a lot!

Are there any specific challenges or areas of improvement related to **efficiency**, **communication**, or **health literacy** in the care of CHD children and their parents that you would like to share? (Open-ended)

Type your answer here

1. Before we go, can we get your **full name** or **ID number**?

Please provide your ID number (sent via email) **or** full name so we can send you incentives.

Your information will be kept confidential and used solely for survey communication.

1. And, what **email address** can we reach you at?

This is only to confirm we received your answers and sent your incentive.

Thank you so much for your time and feedback!

For any questions or feedback, please don't hesitate to contact us.
