# Supplementary material for: Developing Assessments for Key Stakeholders in Pediatric Congenital Heart Disease: Qualitative Pilot Study to Inform Designing of a Medical Education Toy
Source: JMIR Form Res. 2025 Jan 27;9:e63818. doi: 10.2196/63818 (PMC11811657; doi:10.2196/63818)
Supplement: Multimedia Appendix 3 [file formative_v9i1e63818_app3.docx]

## Multimedia Appendix 3

Sample questions and key stakeholders' feedback on survey experience.

| **Categories** | **Sample Questions** (phrased different for stakeholders) | **Stakeholder** | **Remarks (Strength)** | **Remarks (Weakness)** |
| --- | --- | --- | --- | --- |
| Initial Thoughts | 1. What did you think or feel when you first received the survey?  2. Was it easy to move around and understand the survey layout? | Parent (P) | *- "I was totally into it from the start! Seeing kids playing in the picture was a blast, definitely not sad!" - "Layout was clear, and I could follow it easily."* | *- "I didn't think the survey was worth it until I started answering." - "I found one part a bit confusing at first."* |
|  |  | Healthcare Provider (H) | *- "...really interactive with photos and video, and buttons. It's easy…".* | *N/A* |
|  |  | Children (C) | *- "I love the cake, candles! It's so cool!"* | *- "I saw the papers, I thought it's another doctor thingy!"* |
| Understanding the Questions | 3. Were the questions clear and easy to grasp?  4. Did you find any questions difficult to understand or answer? | P | *- "clear, easy, feeling good; finally, somebody asks!"* | **-** *"Maybe I'll change some of the words, they're good but maybe phrase them differently!"* |
|  |  | H | *- "Much better than usual surveys!"* | **-** *"Not really, just change the order to make it more coherent!"* |
|  |  | C | *- "The questions are like painting, playing funny game!"* | **-** *"This one is hard!, mom do you know that?" - " Just like the smiley face, but I don’t know!"* |
| Relevance of Questions | 5. Did the questions cover topics relevant to your experiences?  6. Were there any options you wished were included? | P | *- "I wish they'd asked me right after we found out about her problem. Would've been really helpful!"* | **-** *"Just sometimes, don't assume we get help. First ask if we get help, and then ask what help…"* |
|  |  | H | *- "You cover it all, reminding me of the problems we should solve, even the ones I forgot to think about. So, let's solve them!"* | *- "separate follow-up and new patients. The timing's different, and there's other stuff..."* |
|  |  | C | **-** *"This is fun, just ask more, like asking about my school!"* | **-** *"Can I do more questions? And, do I get a present at the end?"* |
| Completeness of Options | 7. Did the options to answer each question cover the full range of possible responses?  8. Are there any specific options you think should be added? | P | *"You think like us, adding whatever may be possible. Do you have anyone with this problem? your child?"* | *- "Yeah, just add 'none' options to some questions too. I see you have it in most places, but just saying!"* |
|  |  | H | *- "What you're doing here [Matrix Question] is great. Try to have more."* | *- "Ask for the most vulnerable families than general ones…"* |
|  |  | C | *N/A* | *- "Yeah, just add 'none' to some questions too. I see you have it in most places, but just saying!* |
| Survey Length and Time | 9. How did you feel about the length of the survey?  10. Was it hard to find time to complete the survey? | P | *- "Not long, keep us good busy!" - "was like a therapy!" - all felt similar* | *N/A* |
|  |  | H | *- "I thought the length of the survey was appropriate." - all said the same* | *N/A* |
|  |  | C | **-** *"Not too much" - all felt like that* | *N/A* |
| Overall Experience | 11. On a scale of (1 to 10), how would you rate your experience with the survey?  12. Any suggestions to make the survey experience better? | P | *3 parents said 10, and 1 said 8 or 9.* | *- "Thank you for doing that, we're thankful. Why are you thanking us? We need more of this!"* |
|  |  | H | *1 healthcare provider said 10, the other one said 9, and we didn't ask this from the other provider.* | *- "I don’t know if it is helpful, but perhaps have a space for a comment to elaborate on answers to the questions? Otherwise, I wouldn’t change the content at all."* |
|  |  | C | *We asked how much they liked it. They said, " Love it so much," "Loved it, it was fun!", "I had fun," "Can I do it next time again," "I Love it."* | *- " I want to do this more, it was short!" - " Can you stay here, stay, stay, don’t go"* |
